# *Rugulopteryx*-Derived Spatane, Secospatane, Prenylcubebane and Prenylkelsoane Diterpenoids as Inhibitors of Nitric Oxide Production

**DOI:** 10.3390/md21040252

**Published:** 2023-04-19

**Authors:** Belén Cuevas, Ana I. Arroba, Carolina de los Reyes, Eva Zubía

**Affiliations:** 1 Departamento de Química Orgánica, Facultad de Ciencias del Mar y Ambientales, Universidad de Cádiz, 11510 Puerto Real (Cádiz), Spain; belen.cuevas@inibica.es (B.C.); carolina.dereyes@uca.es (C.d.l.R.); 2 Unidad de Investigación, Instituto de Investigación e Innovación Biomédica de Cádiz (INiBICA), Hospital Universitario Puerta del Mar, Avda. Ana de Viya 21, 11009 Cádiz, Spain; anaarroba@gmail.com; 3 Departamento de Biomedicina, Biotecnología y Salud Pública, Facultad de Medicina, Universidad de Cádiz, Pl. Falla, 9, 11003 Cádiz, Spain

**Keywords:** diterpenoids, brown algae, *Rugulopteryx okamurae*, anti-inflammatory, NO inhibition

## Abstract

This study aimed to evaluate the anti-inflammatory potential of the different classes of diterpenoids produced by algae of the genus *Rugulopteryx*. First, sixteen diterpenoids (**1**–**16**), including spatane, secospatane, prenylcubebane, and prenylkelsoane metabolites, were isolated from the extract of the alga *Rugulopteryx okamurae* collected at the southwestern Spanish coasts. Eight of the isolated diterpenoids are new compounds whose structures were determined by spectroscopic means: the spatanes okaspatols A-D (**1**–**4**); the secospatane rugukamural D (**8**); the prenylcubebanes okacubols A (**13**) and B (**14**); and okamurol A (**16**), which exhibits an unusual diterpenoid skeleton featuring a kelsoane-type tricyclic nucleus. Second, anti-inflammatory assays were performed on microglial cells Bv.2 and macrophage cells RAW 264.7. Compounds **1**, **3**, **6**, **12**, and **16** caused significant inhibition of the NO overproduction induced by LPS in Bv.2 cells, and compounds **3**, **5**, **12**, **14**, and **16** significantly decreased levels of NO in LPS-stimulated RAW 264.7 cells. The most active compound was okaspatol C (**3**), which completely suppressed the effects of LPS stimulation, both in Bv.2 and in RAW 264.7 cells.

## 1. Introduction

The genus *Rugulopteryx* of brown algae was described in 2006 by De Clerck et al. to accommodate the species *Dictyota radicans*, *Dilophus suhrii*, and *Dilophus marginatus*, which were removed from *Dictyota* and *Dilophus* genera and, consequently, renamed as *Rugulopteryx radicans*, *R. shurii*, and *R. marginata* [1]. Later, in 2009, the species known as *Dilophus okamurae* was also transferred to the genus *Rugulopteryx* [2]. Although these four species of macroalgae typically grow along several areas of the Pacific Ocean [3], in recent years, *R. okamurae* has become renowned for having severely invaded the coasts of the Strait of Gibraltar, which connect the Mediterranean Sea and the Atlantic Ocean [4].

**Figure 1:**
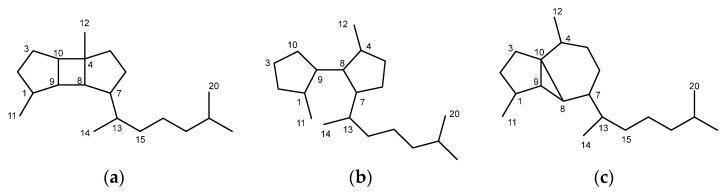
Three characteristic carbon skeletons of diterpenoids of algae of the genus *Rugulopteryx*: (**a**) spatane; (**b**) secospatane; (**c**) prenylcubebane.

Chemical studies of the genus *Rugulopteryx*, performed on *R. marginata* from Australian coasts [5] and on several collections of *R. okamurae* from Japanese [6,7,8,9,10,11,12,13] and southern Spanish coasts [14,15], have shown that these algae are prolific sources of diterpenoids which belong to various structural classes. A group is formed by diterpenoids exhibiting the carbon skeleton of spatane, which contains a fused 5/4/5 tricyclic ring system (Figure 1) [5,8,12,13,14]. A second, more numerous and distinctive group of *Rugulopteryx* diterpenoids is formed by the secospatanes, currently including more than twenty compounds [5,9,10,11,12,13,14,15], which are formally derived from spatanes by cleavage of the bond at C-4,C-10. A third characteristic group comprises four compounds exhibiting a prenylcubebane framework, characterized by possessing a fused 5/3/6 tricyclic ring system [5,7,10,14]. It is noteworthy that secospatane diterpenoids have been exclusively described from algae of the genus *Rugulopteryx*. The only exception is a secospatane reported from an alga identified as *Dictyota fenestrata* [16], which on a chemotaxonomic basis, suggests that the classification of this species might need a revision. On the other hand, spatane diterpenoids have been isolated from other brown algae of the Family Dictyotaceae, such as *D. fenestrata* [16], *Spatoglossum schmittii* [17,18], *S. howleii* [18], and *Stoechospermum marginatum* [19,20,21,22,23]. More recently, four spatanes have also been described from soft corals [24,25,26]. Regarding the prenylcubebanes, besides *Rugulopteryx* algae, the gorgonians *Anthogorgia* sp. and *Euplexaura* sp. have yielded terpenoids displaying this skeleton, although the four compounds isolated from these gorgonians were named as serrulatane-type diterpenoids [27,28]. In addition, the species *R. okamurae* has been the source of a few unique compounds with unprecedented diterpenoid skeletons, such as the dictyterpenoids isolated from Japanese specimens [13] and rugukadiol A from Spanish specimens of the alga [15].

The noticeable structural diversity and unusual frameworks of the diterpenoids produced by algae of the genus *Rugulopteryx* offer great opportunities in the search for leads for the development of novel molecules for biomedical applications. In this regard, we have recently reported the first account on the anti-inflammatory activity of compounds of the secospatane class, in particular, of ruguloptone A and also of the uncommon diterpenoid rugukadiol A obtained from *R. okamurae* collected in the Spanish coasts [15]. These results prompted us to investigate the properties of diterpenoids of the spatane and prenylcubebane classes, whose potential remained unexplored, and to test additional secospatane metabolites. Herein we describe the isolation from *R. okamurae* of further sixteen diterpenoids. The isolated metabolites include seven spatanes (**1**–**7**), five secospatanes (**8**–**12**), and three prenylcubebanes (**13**–**15**), among which okaspatols A–D (**1**–**4**), rugukamural D (**8**), okacubols A (**13**), and B (**14**) are new compounds. In addition, the new okamurol A (**16**), which possesses an unusual kelsoane-type tricyclic nucleus, was also isolated. The capability of diterpenoids of the four structural classes to modulate the production of inflammatory mediators, such as nitric oxide (NO), was assayed. In particular, the anti-inflammatory effects were tested in a classic proinflammatory environment by LPS-stimulated microglial cells Bv.2 and macrophage cells RAW 264.7.

## 2. Results and Discussion

**Figure 2:**
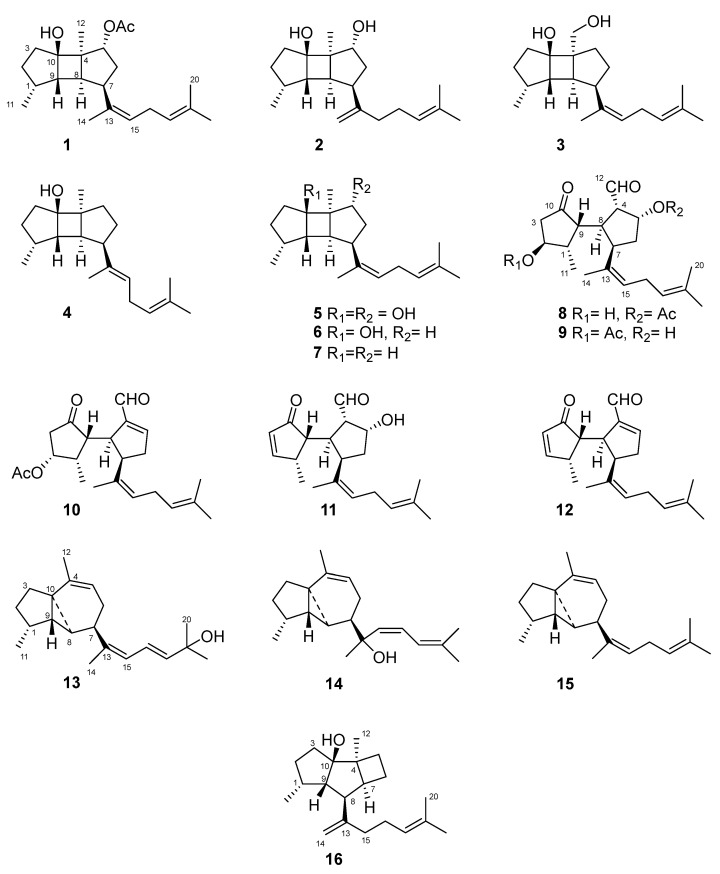
Chemical structures of the diterpenoids isolated from *R. okamurae*.

Fresh specimens of *R. okamurae* were treated with acetone/MeOH, and after evaporation of the solvent, the aqueous residue was extracted with diethyl ether. Column chromatography (CC) of the extract, followed by repeated CC and HPLC purification of selected fractions, led to the isolation of compounds **1**–**16** (Figure 2).

### 2.1. Spatane Diterpenoids ***1***–***7***

Okaspatol A (**1**) possesses the molecular formula C_22_H_34_O_3_, determined by HRESIMS. The IR spectrum showed absorption bands at 3387 and 1711 cm^−1^ due to hydroxy and carbonyl functions, respectively. In particular, the presence of an acetate group was defined from the ^1^H NMR signal at δ 2.03 (3H, s) and the ^13^C NMR signals at δ 172.6 (-COO-) and δ 21.2 (CH_3_-) (Table 1). Another five methyl groups in the ^1^H NMR spectrum (δ_H_ 1.76, 1.67, 1.63, 0.94, 0.82) and the remaining twenty signals of the ^13^C NMR spectrum suggested a diterpene framework for compound **1**.

**Figure 3:**
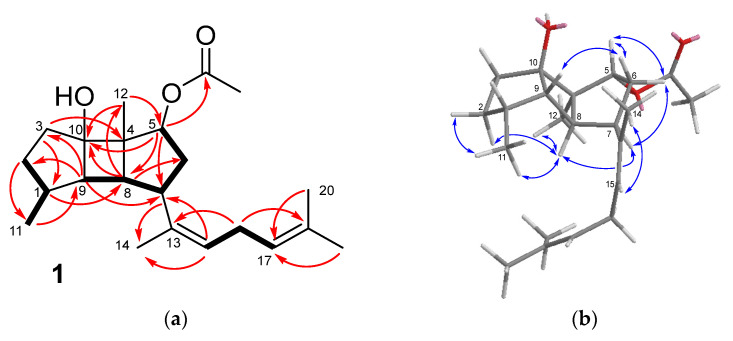
(**a**) Key COSY (bold bond) and HMBC correlations (arrow) observed for compound **1**. (**b**) Key NOESY correlations observed for compound **1**.

The NMR spectra showed the signals of two trisubstituted double bonds (δ_C_ 135.0 (C-13) and δ_C_ 127.3 (C-15)/δ_H_ 5.21 (H-15), δ_C_ 124.5 (C-17)/δ_H_ 5.07 (H-17) and δ_C_ 131.8 (C-18)) and one *bis*-allylic methylene (δ_H_ 2.70, H-16), which together with three methyl groups (δ_H_ 1.76, 1.67, and 1.63) were accommodated in a 1,5-dimethylhexa-1,4-dien-1-yl side chain (Figure 3a). The double bonds and the acetate group mentioned above accounted for three of the six unsaturations deduced from the molecular formula, and hence, compound **1** must be tricyclic. In particular, the presence of a tricarbocyclic core consisting of two five-membered rings fused to a four-membered one, such as that of the spatane diterpenoids, was proposed from the deshielded carbons at δ_C_ 52.2 (C, C-4), 49.6 (CH, C-9), and 44.0 (CH, C-8), which together with the oxygenated carbon at δ_C_ 82.4 (C-10), were attributable to the four bridgehead carbons. Moreover, the methyl groups at δ_H_ 0.82 (d, *J* = 6.6 Hz, Me-11) and 0.94 (s, Me-12) were assigned to the characteristic methyl groups linked to C-1 and C-4 of the spatane skeleton.

These proposals were supported, among others, by the HMBC correlations H-8/C-6, C-10, C-12, H-9/C-2, C-3, C-7, Me-11/C-9, and Me-12/C-10 (Figure 3a). The location of the acetoxy group at C-5 was defined from the HMBC correlations of the geminal proton (δ_H_ 5.49, H-5) with C-7, C-8, C-10, and the acetate carbonyl group together with that of the oxygenated methine carbon (δ_C_ 79.9, C-5) with Me-12. On the other hand, the HMBC correlations H-7/C-14 and H-15/C-7 confirmed the location of the side chain at C-7 of the tricyclic nucleus.

The relative configuration of the molecule was defined from the NOESY data (Figure 3b). The correlations H-8/H-2b, Me-12, and H-9/H-6a supported the *cis*-*anti*-*cis* arrangement of the 5/4/5 tricyclic ring system, exhibiting H-8 and Me-12 on one face of the molecule and H-9 and the hydroxy group on the opposite one. Following this, the NOESY correlations of H-8 with H-7 and Me-11 defined the orientation of the side chain and of Me-11. The α orientation of the acetate group at C-5 was assigned on the basis of the multiplicity of H-5, a doublet with *J* = 4.7 Hz, similar to that reported for spatol, whose stereochemistry was secured by X-ray diffraction analysis [17], for its acetate [18] and related compounds [18,19]. Moreover, when the acetoxy group at C-5 was β-oriented, H-5 has been described as a doublet of doublets with *J* = 10 and 8 Hz [19]. The NOESY correlations of H-5 with both methylene protons at C-6, while H-7 was only correlated with H-6b, were also consistent with the opposite orientation between H-5 and H-7. The *Z* configuration of the double bond at C-13,C-15 was defined by the NOESY correlation H-15/Me-14 and the ^13^C NMR chemical shift of Me-14 at δ_C_ 24.0 [29].

Okaspatol B (**2**) possesses the molecular formula C_20_H_32_O_2,_ determined by HRESIMS. The NMR spectra (Table 1) were related to those of compound **1**, although diagnostic differences were observed, including the absence of the signals due to the acetate group, the shielding of the oxymethine (δ_C_ 75.7/δ_H_ 4.17), and the presence of an olefinic methylene (δ_C_ 108.9/δ_H_ 4.82 and 4.76). The analysis of COSY, HSQC, and HMBC spectra confirmed the spatane framework of compound **2** (Figure S29). The presence of a tertiary hydroxy group at C-10 was supported by the HMBC correlations of the oxygenated carbon at δ_C_ 82.4 (C-10) with H-8, H-9, and Me-12, while the location of a secondary hydroxy group at C-5 was supported by the HMBC correlations of the oxymethine proton (δ_H_ 4.17) with C-7, C-8, and C-10. Differing from **1**, the side chain of **2** contained a double bond at C-13,C-14, as indicated by the HMBC correlation of H-7 with the olefinic methylene carbon. The NOESY correlations (Figure S30) and the multiplicity of H-5 (d, *J* = 4.5 Hz) indicated that compound **2** possessed the same relative configuration as **1** in all its chiral centers.

The molecular formula C_20_H_32_O_2_ was determined by HRESIMS analysis of okaspatol C (**3**). Upon comparison with compound **1**, in the ^1^H NMR spectrum of **3** (Table 2) was evident the absence of the signals of the acetate, the oxymethine, and the Me-12 groups, showing, in turn, the signals of an AB system at δ_H_ 3.63 (d, *J* = 10.8 Hz, H-12a) and 3.43 (d, *J* = 10.8 Hz, H-12b) attributable to an isolated oxymethylene. These data suggested that compound **3** was a spatane diterpenoid bearing a primary hydroxy group at C-12. This proposal was confirmed by the HMBC correlations of the oxymethylene protons with C-5, C-8, and C-10 of the spatane tricyclic nucleus (Figure S29). The NOESY correlations (Figure S30) indicated that the relative configuration of compound **3** was identical to that of **1** and **2**.

Okaspatol D (**4**) possessed the molecular formula C_20_H_32_O, determined by HRESIMS. The NMR spectra (Table 2) were related to those of the spatanes discussed above. However, the ^1^H NMR spectrum of **4** did not show any signal attributable to protons geminal to an oxygenated function. In fact, the ^13^C NMR spectrum of **4** only showed a carbon linked to oxygenated function at δ_C_ 82.2, which was identified as C-10 from its HMBC correlations with H-3, H-5, H-8, H-9, and Me-12 (Figure S29). On the other hand, compound **4** was concluded to possess a side chain that differed from that of **1** and **3** by the *E* configuration of the double bond at C-13,C-15, as indicated by the shielding of Me-14 (δ_H_ 1.59/δ_C_ 17.9) [29] and the NOESY correlation of Me-14 with the methylene protons H-16 (Figure S30).

In addition to the new okaspatols A-D (**1**–**4**) described above, the known spatanes **5**–**7** were also isolated. Compounds **5** and **6** have already been found in *R. okamurae* [14]. Compound **7** has been previously described from *R. marginata*, although only partial and not assigned NMR data were reported [5]. The fully assigned NMR data of compound **7** (Table S1), as well as the key COSY, HMBC, and NOESY correlations, are provided in Figures S29 and S30.

### 2.2. Secospatane Diterpenoids ***8***–***12***

Rugukamural D (**8**) possessed the molecular formula C_22_H_32_O_5_, determined by HRESIMS. The NMR spectra (Table 3) showed the signals of an acetate group (δ_H_ 1.96 (3H, s), δ_C_ 20.9 (CH_3_-), δ_C_ 172.1 (-COO-)), and the remaining twenty carbons of the ^13^C NMR spectrum were attributable to a diterpene. In particular, the presence in the spectra of a ketone (δ_C_ 220.4) and an aldehyde group (δ_C_ 201.7/δ_H_ 9.59) suggested that **8** was a secospatane diterpenoid related to dilkamural and rugukamurals A-C [15], which are characterized by containing a five-membered ketone directly linked to a cyclopentanecarbaldehyde moiety. Moreover, according to the NMR spectra, the molecule contained two trisubstituted double bonds, (δ_C_ 135.7 (C-13), δ_C_ 129.8 (C-15)/δ_H_ 5.26 (H-15) and δ_C_ 123.9 (C-17)/δ_H_ 5.09 (H-17), δ_C_ 132.6 (C-18)), a methine linked to the acetoxy group (δ_C_ 78.2 (C-5)/δ_H_ 5.63 (H-5)), and a methine linked to a hydroxy group (δ_C_ 73.5 (C-2)/δ_H_ 4.10 (H-2)).

**Figure 4:**
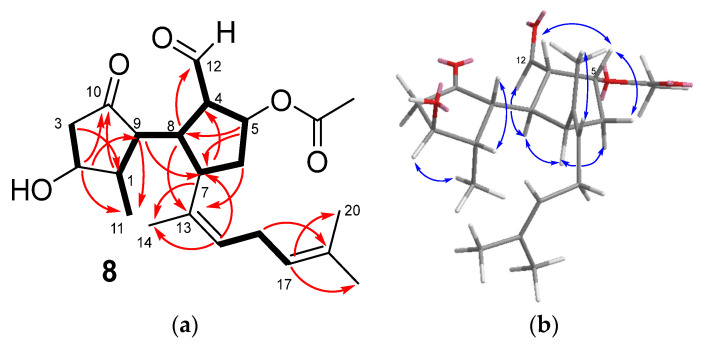
(**a**) Key COSY (bold bond) and HMBC correlations (arrow) observed for compound **8**. (**b**) Key NOESY correlations observed for compound **8**.

On the basis of COSY and HMBC correlations (Figure 4a), the two double bonds mentioned above, together with three methyl groups (δ_H_ 1.69 (Me-14, Me-19), δ_H_ 1.65 (Me-20)) and a *bis*-allylic methylene (δ_C_ 28.1 (C-16)/δ_H_ 2.84, 2.72 (H-16)), were accommodated in a 1,5-dimethylhexa-1,4-dien-1-yl side chain identical to that found in compounds **1**, **3**, and **4**–**7**. The *Z* configuration of the double bond at C-13,C-15 was deduced from the NOESY correlation Me-14/H-15. The position of this chain on the cyclopentanecarbaldehyde ring was supported by the HMBC correlations H-6/C-13, H-7/C-14, H-8/C-13, and H-15/C-7. The HMBC correlation of the proton geminal to the acetoxy group (δ_H_ 5.63, H-5) with C-7 and C-8 determined the presence of the acetoxy group also on this ring, at C-5. On the other hand, the hydroxy group was deduced to be located on the ciclopentanone ring, at C-2, from the HMBC correlations of the proton geminal to the hydroxy group (δ_H_ 4.10, H-2) with C-9, the ketone carbonyl at C-10, and the methyl group at C-11.

The orientation of the aldehyde group, the acetoxy group, H-7, and H-8 on the same side of the ring was deduced from the NOESY correlations H-4/H-5, H-5/H-6a, H-6b/H-7, and H-7/H-8 (Figure 4b). For the other ring, the NOESY correlations H-1/H-9 and H-2/Me-11 indicated that the hydroxy group at C-2 and the Me-11 were on opposite sides of the cyclopentanone ring. These relative configurations were identical to those described for dilkamural [11] and rugukamurals A–C [15]. Based on biogenetic considerations, the stereochemistry of one ring with respect to the other in rugukamural D (**8**) is depicted as identical to that established for dilkamural [11].

Together with rugukamural D (**8**), the four known secospatanes **9**–**12** were also isolated. Compounds **9**–**11** are known diterpenoids of *R. marginata* [5] and herein are described for the first time as metabolites of *R. okamurae*. Compound **12** was first isolated from *R. marginata* [5] and later from *R. okamurae* [13]. Since partial and not assigned NMR data were reported for these four compounds [5], fully assigned ^1^H and ^13^C NMR spectra of **9**–**12**, together with key COSY, HMBC, and NOESY correlations, are provided in Table S2 and Figures S29 and S30.

### 2.3. Prenylcubebane Diterpenoids ***13***–***15***

The molecular formula C_20_H_30_O of okacubol A (**13**) was determined by HRESIMS. This formula, together with the presence of five methyl signals in the ^1^H NMR spectrum at δ_H_ 1.83 (3H), 1.80 (3H), 1.27 (6H), and 1.00 (3H) (Table 4), suggested that **13** was also a diterpenoid. The NMR spectra showed the signals of a tertiary oxygenated carbon (δ_C_ 71.4) and of three double bonds (δ_C_ 143.3,140.4,137.9,126.2,123.0,118.1), bearing in total four olefinic protons (δ_H_ 6.52, 5.83, 5.67, 5.25). Since the three double bonds accounted for three of the six degrees of unsaturation deduced from the molecular formula, compound **13** must be tricyclic.

**Figure 5:**
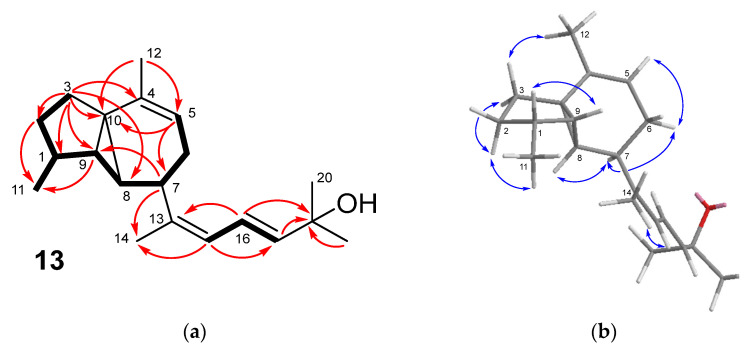
(**a**) Key COSY (bold bond) and HMBC correlations (arrow) observed for compound **13**. (**b**) Key NOESY correlations observed for compound **13**.

Two of the double bonds mentioned above, the oxygenated carbon and three methyl groups, were deduced to be located in a 5-hydroxy-1,5-dimethylhexa-1,3-dien-1-yl side chain. Key data were the HMBC correlations of the oxygenated carbon (δ_C_ 71.4, C-18) with two methyl groups (δ_H_ 1.27, Me-19 and Me-20) and with two olefinic protons (δ_H_ 5.67 (H-17) and 6.52 (H-16)), together with the COSY coupling of these with another olefinic proton (δ_H_ 5.83 (H-15)), which showed allylic coupling with the methyl group at δ_H_ 1.80 (Me-14) (Figure 5a). On the other hand, the remaining methine and methylene groups of the molecule were connected following the sequence of COSY couplings spanning from the olefinic methine proton at δ_H_ 5.25 (H-5) to the methylene at δ_H_ 2.08 (H-3a)/1.74 (H-3b). Then, the HMBC correlations H-3/C-1,C-2,C-9, and C-10 were consistent with a five-membered ring, while the correlation H-3/C-8 defined the presence of the fused three-membered ring. The tricyclic framework was completed from the correlations H-3/C-4, H-5/C-10, and Me-12/C-10. Moreover, the HMBC correlation H-7/C-14 indicated that the side chain was connected to C-7. All these data led to conclude that compound **13** possessed a carbon skeleton of prenylcubebane. The NOESY correlations defined the *cis* orientation of H-1 and H-9 on one side of the molecule and the *cis* orientation of H-7, H-8, and Me-11 on the other side (Figure 5b). The 13*Z*,16*E* configuration of the double bonds of the side chain was deduced from the NOESY correlation Me-14/H-15 and the coupling constant *J*_16,17_ = 15.2 Hz, respectively.

The molecular formula C_20_H_30_O determined by HRESIMS of okacubol B (**14**) indicated that it was an isomer of compound **13**. The analysis of the NMR spectra (Table 4) showed that **14** possessed the same tricyclic core as **13** but a different side chain. In particular, the hydroxy group was deduced to be located at C-13, and the conjugated double bonds at C-15,C-16, and C-17,C-18. Key data were the HMBC correlations of the methyl group at δ_H_ 1.42 (Me-14), both with the oxygenated carbon (δ_C_ 78.4, C-13) and with C-7 in the ring (Figure S29). The 15*Z* configuration was deduced from the coupling constant *J*_15,16_ = 12.1 Hz.

In addition to compounds **13** and **14**, the known prenylcubebane **15** was also isolated. This compound was described from *R. marginata* [5], although only partial NMR data were reported. The full NMR data of compound **15,** together with key COSY, HMBC, and NOESY correlations, are shown in Table S3 and Figures S29 and S30.

### 2.4. Prenylkelsoane Diterpenoid ***16***

**Figure 6:**
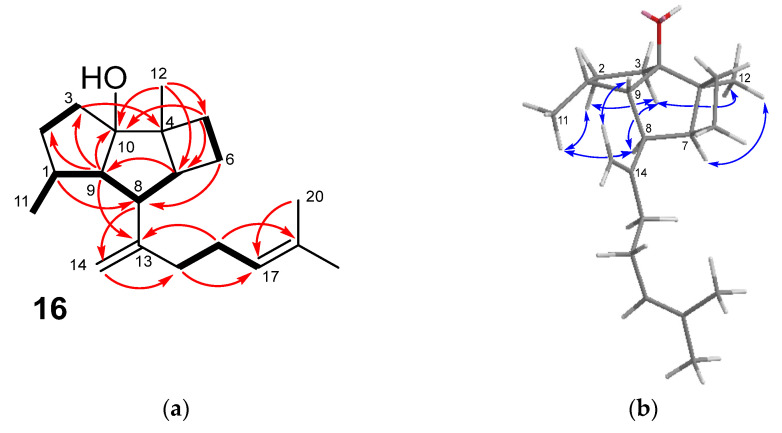
(**a**) Key COSY (bold bond) and HMBC correlations (arrow) observed for compound **16**. (**b**) Key NOESY correlations observed for compound **16**.

The molecular formula C_20_H_32_O of okamurol A (**16**) was determined by HRESIMS, and the IR absorption band at 3384 cm^−1^ indicated the presence of a hydroxy group. The NMR spectra (Table 5) showed the signals of two double bonds, one 1,1-disubstituted (δ_C_ 150.0 (C-13) and δ_C_ 110.5 (C-14)/δ_H_ 4.94,4.85 (H-14)) and one trisubstituted (δ_C_ 125.6 (C-17)/δ_H_ 5.08 (H-17) and δ_C_ 132.2 (C-18)), which together with two allylic methylenes (δ_C_ 37.7(C-15)/δ_H_ 1.91,1.84 (H-15) and δ_C_ 27.4 (C-16)/δ_H_ 2.09,2.05 (H-16)) and two methyl groups (δ_H_ 1.65 (Me-19), δ_H_ 1.59 (Me-20)) were accommodated in a 6-methylhepta-1,5-dien-2-yl side chain similar to that found in **2**. Eliminating these eight carbons and two unsaturations from the molecular formula showed that the remaining twelve carbons of the molecule must be arranged in a tricyclic moiety. In fact, similar to the spatane **4**, the NMR data indicated that the tricyclic core of **16** contained four methylenes, four methines, and two fully substituted carbons, one of them linked to the hydroxy group. However, a careful analysis of the COSY and HMBC correlations (Figure 6a) indicated that **16** did not exhibit the fused 5/4/5 tricyclic ring system characteristic of the spatanes, but an alternative 5/5/4 arrangement of kelsoane type [30], featuring a cyclopentane ring and a cyclobutane ring fused to a central cyclopentane ring.

In particular, the presence of a methyl-substituted five-membered ring was supported by the HMBC correlations of the methine proton at δ_H_ 2.59 (H-9), with two methylene carbons (δ_C_ 32.2 (C-2) and 34.4 (C-3)) and with the oxygenated carbon (δ_C_ 94.6, C-10), together with the sequence of COSY couplings of protons δ_H_ 2.59 (H-9)/δ_H_ 2.50 (H-1)/δ_H_ 0.92 Me-11. Key data to define the central five-membered ring were the COSY sequence of couplings δ_H_ 2.59 (H-9)/δ_H_ 2.38 (H-8)/δ_H_ 2.29 (H-7) together with the HMBC correlation of H-7 with C-9 and of Me-12 with C-7 and C-10. Moreover, the location of the side chain linked to this central ring was deduced from the HMBC correlation of H-9 with the olefinic carbon at δ_C_ 150.0 (C-13). The presence of the four-membered ring was deduced from two COSY-coupled methylenes (δ_H_ 2.05, 1.40 (CH_2_-5) and δ_H_ 1.89, 1.52 (CH_2_-6)), which showed HMBC correlations with the oxygenated carbon C-10 and with the carbon C-8 bearing the side chain, respectively. All these data led to defining that compound **16** possessed a carbon skeleton of prenylkelsoane. The relative configuration of compound **16** was determined from the NOESY data. Thus, the correlation H-7/Me-12 indicated the *cis* fusion between the four- and five-membered rings (Figure 6b). The correlations of H-3a with H-8, Me-12, H-2b, and H-2b and H-8 with Me-11 indicated that H-8, Me-11, and Me-12 were on the same face of the molecule. On the other face were H-9 and the side chain, as supported by the NOESY correlation between H-9 and H-14. Although the absolute configuration has not been determined, compound **16** has been depicted on the basis of biogenetic considerations (see below), with the same configuration as the spatanes and other diterpenoids isolated from this alga.

**Figure 7:**
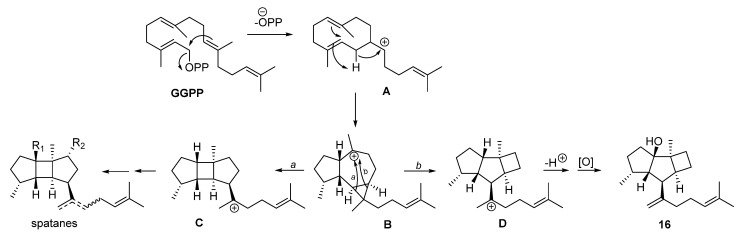
Possible biosynthetic pathway to spatanes and prenylkelsoane **16** in *Rugulopteryx*.

The prenylkelsoane carbon skeleton exhibited by compound **16** is very unusual. A survey of the literature revealed only one previous account, which describes the isolation of a deoxyanalogue of **16** from the mixture resulting from the incubation of geranylgeranyl diphosphate (GGPP) with a terpene synthase isolated from the marine bacterium *Streptomyces xinghaiensis* [31]. It is worth noting that in that study, the diterpene spata-13,17-diene was the major compound derived from GGPP, leading to the proposal of the formation of the tricyclic core of spatanes and prenylkelsoanes from a common biosynthetic intermediate, which was supported by isotope labeling experiments [31]. The biosynthesis of spatanes and the prenylkelsoane okamurol A (**16**) in the alga *Rugulopteryx* could take place in a similar way, as shown in Figure 7. After the cyclization of GGPP to yield the (*E*,*E*)-prenylgermacradienyl cation **A**, a second cyclization would produce cation **B**. This could experience two alternative openings of the cyclopropane ring to give **C** and **D**, which are precursors of the spatanes and of the prenylkelsoane **16**, respectively.

### 2.5. Inhibitory Activity of NO Production

The anti-inflammatory activity of ten of the isolated compounds, including representatives of the four classes of diterpenoids, was assayed in specific immune cell lines, microglial cells Bv.2, and macrophage cells RAW 264.7. In particular, the capability of the spatane diterpenoids **1**, **3**, **5**, and **6**, the secospatanes **8**, **10**, **11**, and **12**, the prenylcubebane **14**, and the prenylkelsoane **16** to inhibit the lipopolysaccharide(LPS)-stimulated production of nitric oxide (NO) in Bv.2 cells and RAW 264.7 cells was tested. Compounds **7**, **9**, and **13** were not tested because of solubility issues or low stability, while compounds **2** and **4** were expected to cause similar effects to compounds **5** and **6**.

First, in order to select the highest dose of each compound that could be used in the assays without affecting cell viability, the cytotoxicity of each diterpenoid against Bv.2 and RAW 264.7 cells was evaluated (Figures S31 and S32). The spatane diterpenoids **1**, **3**, **5**, **6,** and the prenylkelsoane **16** were the less toxic compounds, and in most instances, they did not affect cell viability at concentrations up to 10 μM or even 25 μM for compound **3**. On the other hand, the secospatanes **8**, **10**, **11**, and **12** were much more toxic for both microglial and macrophage cells, and at 10 μM caused more than 70% of cell death for Bv.2 and more than 60% of cell death for RAW 264.7. The prenylcubebane **14** did not affect Bv.2 cells viability at concentrations up to 1 μM and RAW 264.7 up to 10 μM. On this basis, the highest doses of the compound used in the NO inhibitory assays with Bv.2 and RAW 264.7 cells were those shown in Table 6 in order to guarantee that decreases of NO levels in the cells were truly due to inhibition of its production and not to cell death.

**Figure 8:**
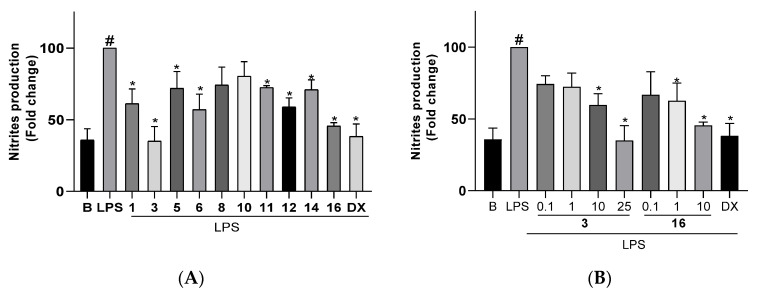
(**A**) Effects of diterpenoids **1** (10 μM), **3** (25 μM), **5** (10 μM), **6** (10 μM), **8** (0.1 μM), **10** (0.5 μM), **11** (0.5 μM), **12** (0.1 μM), **14** (1 μM), and **16** (10 μM) on NO release in microglial cells Bv.2. (**B**) Dose-response effects of diterpenoids **3** (0.1, 1, 10, and 25 μM ) and **16** (0.1, 1, 10 μM ) on NO release in microglial cells Bv2. Bv.2 cells were pretreated for 3 h with the compound at the corresponding concentration or with dexamethasone (DX, 2.5 μM), followed by stimulation with 200 ng/mL LPS for 24 h. Nitrites accumulation in the culture media was measured using the Griess reagent. Results are expressed as fold change relative to the LPS condition and are mean ± SD (n ≥ 3 independent experiments performed in duplicate). Significant differences were determined by two-way ANOVA followed by Bonferroni *t*-test. * *p* ≤ 0.05 vs. LPS. ^#^ *p* ≤ 0.05 vs. Basal.

**Figure 9:**
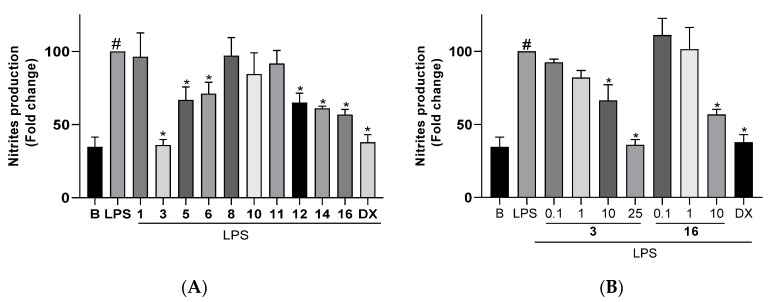
(**A**) Effects of diterpenoids **1** (1 μM), **3** (25 μM), **5** (10 μM), **6** (10 μM), **8** (0.1 μM), **10** (0.5 μM), **11** (0.5 μM), and **12** (1 μM), **14** (10 μM), and **16** (10 μM) on NO release in macrophage cells RAW 264.7 (**B**) Dose-response effects of diterpenoids **3** (0.1, 1, 10, and 25 μM ) and **16** (0.1, 1, 10 μM) on NO release in RAW 264.7 cells. RAW 264.7 cells were pretreated for 3 h with the compound at the corresponding concentration or with dexamethasone (DX, 2.5 μM), followed by stimulation with 200 ng/mL LPS for 24 h. Nitrite accumulation in the culture media was measured using the Griess reagent. Results are expressed as fold change relative to the LPS condition and are mean ± SD (n ≥ 3 independent experiments performed in duplicate). Significant differences were determined by two-way ANOVA followed by Bonferroni *t*-test. * *p* ≤ 0.05 vs. LPS. ^#^ *p* ≤ 0.05 vs. Basal.

For the anti-inflammatory assays, cells were pretreated for 3 h with the compounds at the highest non-cytotoxic doses previously evaluated (Table 6) and then stimulated with LPS (200 ng/mL) to trigger an inflammatory response, which includes the overproduction and release of NO. The production of this inflammatory mediator was determined by measuring the levels in the culture medium of nitrite secreted by the cells, which is one of the major metabolites derived from NO. The treatment of cells with the compounds in the absence of LPS did not cause any induction of inflammatory response. The changes observed in NO production in microglial cells Bv.2 and macrophage cells RAW 264.7 after pretreatment with the compounds and LPS stimulation are shown in Figure 8 and Figure 9, respectively.

Figure 8A shows the nitrite production measured in cultures of control Bv.2 cells (B bar), in cultures of cells stimulated with LPS (LPS bar), and in cultures of cells treated with the algal diterpenoids at the selected concentration or with dexamethaxone (DX bar) and subsequently stimulated with LPS. Upon stimulation of cells with LPS, the nitrites level increased threefold compared to control Bv.2 cells. However, treatment of cells with diterpenoids **1**, **3**, **5**, **6**, **11**, **12**, **14**, and **16** significantly inhibited nitrite production, with decreases of 38.8%, 65.0%, 28.1%, 43.0%, 27.6%, 41.1%, 29.1%, and 54.4%, respectively, in relation to LPS-stimulated but non-treated cells. The inhibition caused by okaspatol C (**3**) and okamurol A (**16**) was outstanding, since effectively counteracted the effect of LPS stimulation. Upon treatment with these compounds, and in spite of adding the inflammatory stimulus, the level of nitrites remained identical or close to that of basal conditions and similar to the levels observed in cells treated with the classical anti-inflammatory steroid dexamethasone. Both compounds showed dose-dependent inhibitory activity (Figure 8B). The strongest inhibition was caused by compound **3** at 25 μM, although significant effects were also observed at a concentration of 10 μM, with compound **16** being more active than **3**. The spatanes **1** and **6** and the secospatane **12** also caused noticeable inhibitory effects, suppressing the LPS-induced overproduction of nitrites to less than half.

As shown in Figure 9A, the treatment of macrophage cells RAW 264.7 with LPS induced the overproduction of nitrites. The pretreatment of cells with compounds **3**, **5**, **6**, **12**, **14**, and **16** caused inhibition of the nitrite level by 64.0%, 30.8%, 28.9%, 35.0%, 39.0%, and 43.2%, respectively, in relation to LPS-stimulated but non-treated cells. Similar to that observed with Bv.2 cells, compound **3** completely suppressed the effects of LPS stimulation in RAW 264.7 cells. The inhibition of nitrite production caused by compound **16** was also noticeable, although more moderated, suppressing the LPS-induced overproduction of nitrites to less than half. Both compounds, **3** and **16**, showed dose-dependent inhibitory activity (Figure 9B). The strongest inhibition was observed for **3** at 25 μM, while at 10 μM, compound **16** was more active than **3**. The spatane **5**, the secospatane **12**, and the prenylcubebane **14** were also capable of reducing the overproduction of NO to half.

Although diterpenoids of the spatane class have been known for a long time, data on their biomedical properties are scarce and mostly focused on the cytotoxicity shown by a few compounds in assays against cancer cell lines [17,18,25,32,33]. This study has shown the anti-inflammatory properties of various spatane diterpenoids, with okaspatol C (**3**) outstanding because of its capacity to completely inhibit the overproduction of NO induced upon inflammatory stimulation, both in microglial Bv.2 and in macrophage RAW 264.7 cells. On the other hand, most of the compounds of the secospatane series tested in this study showed weak or no significant inhibitory activity. This outcome strengths observations made in our previous study where another six secospatanes were assayed [15]. Altogether, the results suggest that the 1,5-dicarbonyl system present in secospatanes **8**, **10**, **11**, **12**, dilkamural [15], and rugukamural C [15] enhances cytotoxicity, constraining the use of these compounds to very low doses, which may not be effective in NO inhibition. On the other hand, secospatanes, such as ruguloptones A, B, and F, exhibiting a –CH_2_OR group at C-12 instead of the aldehyde function, are not toxic and effective NO inhibitors [15]. NO inhibitory activity was also detected for the prenylcubebane **14** and with more potency for the prenylkelsoane **16**, although only a compound of each of these diterpenoid classes has been tested.

During the last three decades, growing evidence has shown that enhanced NO production, due to the expression of inducible nitric oxide synthase (iNOS), plays a key role in the pathophysiology of inflammation [34,35]. Thus, inhibition of iNOS is an important strategy to control the inflammatory processes associated with many pathological conditions. In this regard, structurally diverse natural products, mostly isolated from plants, have been reported to act by inhibiting NO production [35]. In recent years, several metabolites of macroalgae, including phenolic compounds [36,37,38], fucosterol [36], fucoxanthin [39], C_15_ acetogenins [40], and terpenoids [40,41,42], have been described to decrease NO levels in LPS-stimulated macrophage RAW 264.7 cells and fucosterol also in microglial cells [43]. Together with our previous report [15], this study has demonstrated the NO inhibitory activity of algal diterpenoids of four structural classes in both immune cells Bv.2 and RAW 264.7 cells, showing the potential of macroalgal terpenoids in the search for lead compounds for new anti-inflammatory agents.

## 3. Materials and Methods

### 3.1. General Experimental Procedures

Optical rotations were measured on a Jasco P-2000 polarimeter (Jasco, Easton, MD, USA). IR spectra were recorded on a Perkin–Elmer FT-IR Spectrum Two spectrometer (Perkin Elmer, Boston, MA, USA). ^1^H and ^13^C NMR spectra were recorded on an Agilent 500 (Agilent Technologies, Santa Clara, CA, USA) or on a Bruker 500 spectrometer (Bruker, Billerica, MA, USA), using CD_3_OD as solvent. Chemical shifts were referenced using the solvent signals at δ_H_ 3.30 and δ_C_ 49.0. COSY, HSQC, HMBC, and NOESY experiments were performed using standard Agilent or Bruker pulse sequences. High-resolution mass spectra (HRESIMS) were obtained on a Waters XEVO G2-S Mass spectrometer (Waters, Milford, MA, USA). Column chromatography was carried out on Merck Silica gel 60 (70–230 mesh) (Merck, Darmstadt, Germany). SPE separations were performed on Supelco DSC18 cartridges (Supelco, Bellefonte, PA, USA). HPLC separations were performed on a LaChrom-Hitachi apparatus (Merck, Darmstadt, Germany) using a differential refractometer RI-71. Luna Si (2) (250 × 4.6 mm, 5 μm) (Phenomenex, Torrance, CA, USA) and Luna Si (2) (250 × 10 mm, 5 μm) (Phenomenex, Torrance, CA, USA) columns were used for separations in normal phase. All solvents were of HPLC grade.

### 3.2. Algae Collection

Specimens of *R. okamurae* (E.Y. Dawson), I. K. Hwang, W. J. Lee, and H. S. Kim (Class Phaeophyceae, Order Dictyotales, Family Dictyotaceae) were collected at Punta Carnero (Cádiz, Spain, 36°04′ 38.6′′ N; 5°25′31.1′′ W) and transported to the laboratory in a thermal refrigerator. Algae were washed with fresh water to remove epiphytes and organic and inorganic debris and immediately extracted. A voucher specimen (RO-1019) is deposited at the Marine Natural Products Laboratory, Faculty of Marine and Environmental Sciences, University of Cadiz, Spain.

### 3.3. Extraction and Isolation

Fresh samples of *R. okamurae* (500 g) were extracted, and the extract was subjected to silica gel column chromatography, as described previously [15]. In brief, the algae were extracted with acetone/MeOH (1:1, *v*/*v),* and after evaporation of the solvent, the aqueous residue was extracted with Et_2_O. The Et_2_O extract (8.2 g) was subjected to silica gel column chromatography using hexanes/Et_2_O mixtures, Et_2_O, CHCl_3_/MeOH (8:2, *v*/*v),* and finally, MeOH. The fraction eluted with hexanes/Et_2_O (9:1, *v/v*) was further separated by column chromatography (*n*-hexane/Et_2_O mixtures 99:1 to 9:1, *v/v*), and the subfractions showing in their NMR spectra signals attributable to terpenoids were subjected to HPLC (*n*-hexane/EtOAc,99:1, *v/v*) yielding compounds **7**, **14**, and **15**. Further separation of the fraction that eluted with hexanes/Et_2_O (8:2, *v/v*) by column chromatography (*n*-hexane/Et_2_O mixtures 99:1 to 8:2, *v/v*) and HPLC (*n*-hexane/EtOAc, 95:5, *v/v*) of selected subfractions yielded compound **4** and further amounts of **14**. The fraction that eluted with hexanes/Et_2_O (7:3, *v/v*) was subjected to silica gel column chromatography (*n*-hexane/Et_2_O 95:5 to 6:4, *v/v*) and selected subfractions separated by HPLC (*n*-hexane/EtOAc, 95:5 to 8:2 *v/v*) to yield compounds **1**, **6, 10**, **11**, **12**, **13**, and **16**. The fraction eluted with hexanes/Et_2_O (3:7, *v/v*) was separated on a silica gel column (*n*-hexane/Et_2_O mixtures, 8:2 to 1:1, *v/v*) and then purified by HPLC (*n*-hexane/EtOAc 7:3 *v/v*) yielding compound **2**. The fraction that was eluted with Et_2_O was purified on SPE-C18 cartridges (1 g/6 mL) preconditioned with MeOH/H_2_O (9:1, *v*/*v*, 2 mL) and eluted with 10 mL of the same solvent. After evaporation of the solvent, the resulting mixture was separated by silica gel column chromatography (*n*-hexane/Et_2_O mixtures, 75:25 to 1:1, *v/v,* and AcOEt), and selected subfractions were further purified by HPLC (*n*-hexane/EtOAc 7:3 *v/v*) yielding compounds **5**, **8, 9,** and further amounts of **2**. The fraction eluted with CHCl_3_/MeOH was separated on a silica gel column using *n*-hexane/Et_2_O mixtures (6:4 to 4:6, *v/v*) followed by HPLC (*n*-hexane/EtOAc, 1:1 *v/v*) of selected fractions to yield compound **3**. The total amounts obtained of each compound were as follows: **1** (5.7 mg); **2** (4.6 mg); **3** (10.7mg); **4** (8.6 mg); **5** (52.2 mg); **6** (23.3 mg); **7** (10.0 mg); **8** (8.0 mg); **9** (15.6 mg); **10** (23.1 mg); **11** (18.1 mg); **12** (22.7 mg); **13** (7.7 mg); **14** (9.9 mg); **15** (35.3 mg); **16** (9.4 mg).

### 3.4. Characterization of Compounds

Okaspatol A (**1**): colorless oil; ${\left[\alpha \right]}_{D}^{25}$ +34.5 (*c* 0.05, MeOH); IR (film) υ_max_ 3387, 2943, 2866, 1711, 1452 cm^−1^; ^1^H NMR (CD_3_OD, 500 MHz) and ^13^C NMR (CD_3_OD, 125 MHz), Table 1; HRESIMS *m*/*z* 369.2409 [M + Na]^+^ (calcd. for C_22_H_34_O_3_Na 369.2406).

Okaspatol B (**2**): colorless oil; ${\left[\alpha \right]}_{D}^{25}$ +13.7 (*c* 0.12, MeOH); IR (film) υ_max_ 3391, 2931, 2864, 1448 cm^−1^; ^1^H NMR (CD_3_OD, 500 MHz) and ^13^C NMR (CD_3_OD, 125 MHz), Table 1; HRESIMS *m*/*z* 327.2315 [M + Na]^+^ (calcd. for C_20_H_32_O_2_Na 327.2300).

Okaspatol C (**3**): colorless oil; ${\left[\alpha \right]}_{D}^{25}$ +91.6 (*c* 0.09, MeOH); IR (film) υ_max_ 3357, 2953, 2865, 1450 cm^−1^; ^1^H NMR (CD_3_OD, 500 MHz) and ^13^C NMR (CD_3_OD, 125 MHz), Table 2; HRESIMS *m*/*z* 287.2385 [M + H − H_2_O]^+^ (calcd. for C_20_H_31_O 287.2375).

Okaspatol D (**4**): colorless oil; ${\left[\alpha \right]}_{D}^{25}$ +21.6 (*c* 0.07, MeOH); IR (film) υ_max_ 3387, 2942, 2849, 1445 cm^−1^; ^1^H NMR (CD_3_OD, 500 MHz) and ^13^C NMR (CD_3_OD, 125 MHz), Table 2; HRESIMS *m*/*z* 311.2357 [M + Na]^+^ (calcd. for C_20_H_32_ONa 311.2351).

Rugukamural D (**8**): colorless oil; ${\left[\alpha \right]}_{D}^{25}$ +25.8 (*c* 0.13, MeOH); IR (film) υ_max_ 3356, 2931, 2865, 1731, 1240 cm^−1^; ^1^H NMR (CD_3_OD, 500 MHz) and ^13^C NMR (CD_3_OD, 125 MHz), Table 3; HRESIMS *m*/*z* 399.2142 [M + Na]^+^ (calcd. for C_22_H_32_O_5_Na 399.2147).

Okacubol A (**13**): colorless oil; ${\left[\alpha \right]}_{D}^{25}$ +22.9 (*c* 0.11, MeOH); IR (film) υ_max_ 3355, 2946, 2866, 1642, 1595, 1447 cm^−1^; ^1^H NMR (CD_3_OD, 500 MHz) and ^13^C NMR (CD_3_OD, 125 MHz), Table 4; HRESIMS *m*/*z* 309.2211 [M + Na]^+^ (calcd. for C_20_H_30_ONa 309.2194).

Okacubol B (**14**): colorless oil; ${\left[\alpha \right]}_{D}^{25}$ +22.3 (*c* 0.09, MeOH); IR (film) υ_max_ 3460, 2948, 2864, 1648, 1595, 1448 cm^−1^; ^1^H NMR (CD_3_OD, 500 MHz) and ^13^C NMR (CD_3_OD, 125 MHz), Table 4; HRESIMS *m*/*z* 309.2198 [M + Na]^+^ (calcd. for C_20_H_30_ONa 309.2194).

Okamurol A (**16**): colorless oil; ${\left[\alpha \right]}_{D}^{25}$ −26.9 (*c* 0.11, MeOH); IR (film) υ_max_ 3384, 2964, 1234 cm^−1^; ^1^H NMR (CD_3_OD, 500 MHz) Table 5; ^13^C NMR (CD_3_OD, 125 MHz), Table 5; HRESIMS *m*/*z* 311.2349 [M + Na]^+^ (calcd. for C_20_H_32_ONa 311.235126).

### 3.5. Cell Culture

Mouse microglia Bv.2 cell line was purchased from AcceGen Biotechnology (Fairfield, NJ, USA). Mouse macrophages RAW 264.7 cell line was supplied by Dr. A. M. Valverde (IIBm “Alberto Sols” UAM-CSIC, Madrid, Spain). Then, 1.5 × 10^5^ cells/well were seeded in a 6-multiwell plate (Sarstedt, Germany). The culture conditions were 37 °C in a humidified atmosphere with 5% CO_2_ in RPMI supplemented with 10% (*v/v*) heat-inactivated Fetal Bovine Serum (FBS), 1% (*v/v*) penicillin/streptomycin (Sigma), and 2 mM L-glutamine (Gibco, Carlsbad, CA, USA). All experimental cell approaches were performed in a complete medium without FBS.

### 3.6. Analysis of the Cellular Viability by Crystal Violet Staining

Cells were cultured in 24-well plates and grown up to 70% confluence. The cells were treated with solutions of the diterpenes to reach final concentrations of 0.1, 1.0, 10.0, 25.0, and 50.0 µM and incubated in a serum-free medium. After 24 h, the medium was discarded, and cells were fixed by adding 0.5 mL of glutaraldehyde 1% (*v/v*) for 30 min. Then, the plates were rinsed with Phosphate Buffer Saline (PBS), and the remaining viable adherent cells were stained with crystal violet 0.1% (*w/v*) for 30 min. After rinsing plates with water and drying for 24 h, 0.5 mL of acetic acid 10% (*v/v*) was added. The absorbance of each plate was read spectrophotometrically at 590 nm in a microplate reader (Versamax Tunable Microplate reader, Molecular Devices, Sunnyvale, CA, USA).

### 3.7. Analysis of Nitrites (NO_2_^−^)

Cells were cultured in 6-well plates and grown up to 70% confluence. The cells were pre-treated for 3 h with the diterpenes at the corresponding concentration in serum-free medium and then stimulated with lipopolysaccharide (LPS, 200 ng/mL) for another 24 h. Dexamethasone (Dx) was used as a positive reference compound at 2.5 µM. After cell treatments, levels of nitrites were measured by using the Griess reaction assay [44]. Briefly, the cell-cultured medium was treated with an acid solution containing 1% sulphanilamide and 0.1% *N*-(1-naphthyl) ethylenediamine (NEDA) and read spectrophotometrically at 548 nm in a microplate reader.

### 3.8. Statistical Analysis

Data are presented as mean ± standard deviation (SD) and were compared by using the Bonferroni ANOVA test. All statistical analyses were performed using the GraphPad Prism 8.0 software (GraphPad Software Inc., San Diego, CA, USA) with 2-sided tests. Differences were considered statistically significant at *p* ≤ 0.05.

## 4. Conclusions

The brown alga *Rugulopteryx okamurae*, which expands along the coasts of the Strait of Gibraltar, is a rich source of diterpenoids exhibiting spatane, secospatane, and prenylcubebane carbon skeletons. The results herein obtained, together with data from the literature, suggest that the coexistence of these three diterpenoid classes is a characteristic of algae of the genus *Rugulopteryx*. In addition, *R. okamurae* contains okamurol A (**16**), which displays an uncommon diterpenoid skeleton with a 5/5/4 tricarbocyclic nucleus, likely derived from the same biosynthetic precursor of spatanes through an alternative ring closure reaction. From a biomedical point of view, several diterpenoids produced by *Rugulopteryx* are capable of inhibiting the production of the inflammatory mediator NO, with compounds such as the spatane okaspatol A (**3**) and the prenylkelsoane okamurol A (**16**) causing strong suppressive effects of NO overproduction in LPS-stimulated microglial cells Bv.2 and macrophage cells RAW264.7.

## Figures and Tables

**Table 1:** NMR data of the spatane diterpenoids **1** and **2** in CD_3_OD ^a,b^.

Position	1		2	
	δ_C_, Type	δ_H_, m (*J* in Hz)	δ_C_, Type	δ_H_, m (*J* in Hz)
1	36.6, CH	2.12, m	36.8, CH	2.06, m
2	34.5, CH_2_	1.78, m 1.28, m	34.7, CH_2_	1.75, m 1.29, m
3	38.4, CH_2_	2.08, ddd (13.2,13.2,6.2) 1.51, ddd (13.2,13.2,6.9)	38.7, CH_2_	2.08, ddd (13.1,13.1,6.4) 1.48, ddd (13.1,13.1,6.8)
4	52.2, C		52.9, C	
5	79.9, CH	5.49, d (4.7)	75.7, CH	4.17, d (4.5)
6	37.0, CH_2_	2.46, ddd (13.6,13.6,4.7) 1.78, m	38.6, CH_2_	2.26, ddd (13.3,13.3,4.5) 1.64, m
7	42.7, CH	3.19, ddd (13.6,5.9,5.9)	46.4, CH	2.97, ddd (13.3,5.5,5.5)
8	44.0, CH	1.84, m	41.4, CH	1.72, m
9	49.6, CH	2.15, m	48.2, CH	1.87, br dd (6.4,4.5)
10	82.4, C		82.4, C	
11	14.5, CH_3_	0.82, d (6.6)	15.1, CH_3_	0.80, d (6.8)
12	13.6, CH_3_	0.94, s	14.0, CH_3_	1.02, s
13	135.0, C		149.5, C	
14	24.0, CH_3_	1.76, d (1.1)	108.9, CH_2_	4.82, s; 4.76, s
15	127.3, CH	5.21, br t (7.2)	37.8, CH_2_	1.95, m
16	27.8, CH_2_	2.70, m	27.7, CH_2_	2.19, m 1.99, m
17	124.5, CH	5.07, br t (7.2)	125.4, CH	5.11, br t (7.0)
18	131.8, C		132.3, C	
19	25.9, CH_3_	1.67, d (1.1)	25.9, CH_3_	1.66, d (1.0)
20	17.8, CH_3_	1.63, br s	17.7, CH_3_	1.60, br s
CH_3_COO-	172.6, C			
CH_3_COO-	21.2, CH_3_	2.03, s		

^a 1^H at 500 MHz. ^13^C at 125 MHz. ^b^ Assignments aided by COSY, HSQC, HMBC, and NOESY experiments.

**Table 2:** NMR data of the spatane diterpenoids **3** and **4** in CD_3_OD ^a,b^.

Position	3		4	
	δ_C_, Type	δ_H_, m (*J* in Hz)	δ_C_, Type	δ_H_, m (*J* in Hz)
1	36.8, CH	2.10, m	37.0, CH	2.06, m
2	34.7, CH_2_	1.77, m 1.28, m	34.5, CH_2_	1.75, m 1.30, m
3	38.1, CH_2_	2.08, m 1.56, m	38.0, CH_2_	2.03, m 1.46, m
4	54.1, C		48.2, C	
5	30.7, CH_2_	2.20, dd (12.8, 6.9) 1.59, m	35.7, CH_2_	2.11, dd (12.7,6.7) 1.25, m
6	30.1, CH_2_	2.10, m 1.72, m	29.4, CH_2_	1.91, m 1.52, m
7	45.5, CH	2.82, ddd (12.7,5.8,5.8)	52.2, CH	2.44, m
8	39.8, CH	1.83, br dd (5.5, 5.2)	41.6, CH	1.71, br dd (5.5,4.8)
9	49.9, CH	2.08, m	48.7, CH	1.80, br dd (5.5, 5.1)
10	81.8, C		82.2, C	
11	14.4, CH_3_	0.81, d (6.4)	15.1, CH_3_	0.79, d (6.7)
12	65.0, CH_2_	3.63, d (10.8) 3.43, d (10.8)	20.7, CH_3_	1.01, s
13	135.9, C		134.6, C	
14	24.2, CH_3_	1.76, d (1.2)	17.9, CH_3_	1.59, br s
15	126.7, CH	5.14, br t (7.0)	123.6, CH	5.17, br t (7.1)
16	27.8, CH_2_	2.71, m 2.65, m	27.9, CH_2_	2.72, m 2.68, m
17	124.7, CH	5.07, br t (6.7)	124.8, CH	5.09, br t (7.2)
18	131.7, C		131.8, C	
19	25.9, CH_3_	1.66, d (1.1)	25.9, CH_3_	1.66, br s
20	17.8, CH_3_	1.61, br s	17.8, CH_3_	1.62, br s

^a 1^H at 500 MHz. ^13^C at 125 MHz. ^b^ Assignments aided by COSY, HSQC, HMBC, and NOESY experiments.

**Table 3:** NMR data of secospatane **8** in CD_3_OD ^a,b^.

Position	δ_C_, Type	δ_H_, m (*J* in Hz)	Position	δ_C_, Type	δ_H_, m (*J* in Hz)
1	43.5, CH	2.50, m	11	14.5, CH_3_	0.92, d (7.4)
2	73.5, CH	4.10, br d (5.6)	12	201.7, CH	9.59, d (2.3)
3	45.1, CH_2_	2.41, dd (19.1,5.6) 2.07, m	13	135.7, C	
4	59.4, CH	3.49, ddd (9.7,7.2,2.3)	14	22.5, CH_3_	1.69, br s
5	78.2, CH	5.63, ddd (7.2,6.6,4.3)m	15	129.8, CH	5.26, br t (7.1)
6	38.1, CH_2_	2.12, m 1.89, ddd (14.2,8.1,4.3)	16	28.1, CH_2_	2.84, ddd (15.5,7.1,7.1) 2.72, ddd (15.7,7.1,7.1)
7	41.7, CH	3.57, m	17	123.9, CH	5.09, br t (7.1)
8	39.3, CH	2.93, ddd (9.8,9.7,9.3)	18	132.6, C	
9	51.4, CH	2.64, dd (9.8,7.3)	19	25.8, CH_3_	1.69, br s
10	220.4, C		20	17.9, CH_3_	1.65, br s
CH_3_COO	172.1, C				
CH_3_COO	20.9, CH_3_	1.96, s			

^a 1^H at 500 MHz.^13^C at 125 MHz. ^b^ Assignments aided by COSY, HSQC, HMBC, and NOESY experiments.

**Table 4:** NMR data of the prenylcubebane diterpenoids **13** and **14** in CD_3_OD ^a,b^.

Position	13		14	
	δ_C_, Type	δ_H_, m (*J* in Hz)	δ_C_, Type	δ_H_, m (*J* in Hz)
1	35.8, CH	2.30, m	35.8, CH	2.27, m
2	30.8, CH_2_	1.68, m 0.88,m	30.8, CH_2_	1.66,m 0.91, m
3	30.7, CH_2_	2.08, ddd (12.3.11.6,8.3) 1.74, dd (12.3,7.8)	30.7, CH_2_	2.07, ddd (12.3,11.5,8.3) 1.73, m
4	137.9, C		137.4, C	
5	118.1, CH	5.25, br d (7.0)	118.2, CH	5.20, br d (7.0)
6	27.5, CH_2_	1.84, m 1.64, m	24.2, CH_2_	2.02, m 1.51, m
7	34.1, CH	3.02, m	44.1, CH	1.84, ddd (9.3,8.7,3.5)
8	24.7, CH	0.98, m	21.49, CH	1.24, m
9	34.9, CH	1.64, m	35.3, CH	1.49, dd (4.6,4.6)
10	32.1, C		32.1, C	
11	18.2, CH_3_	1.00, d (6.6)	18.6, CH_3_	1.03, d (6.5)
12	21.7, CH_3_	1.83, br s	21.52, CH_3_	1.80, br s
13	143.3, C		78.4, C	
14	20.5, CH_3_	1.80, br d (1.0)	28.1, CH_3_	1.42, s
15	126.2, CH	5.83, br d (10.9)	134.0, CH	5.38, d (12.1)
16	123.0, CH	6.52, dd (15.2,10.9)	126.2, CH	6.18, dd (12.1,11.7)
17	140.4, CH	5.67, d (15.2)	123.4, CH	6.72, br d (11.7)
18	71.4, C		136.2, C	
19	30.09 ^c^, CH_3_	1.27, s	26.6, CH_3_	1.78, br s
20	30.07 ^c^, CH_3_	1.27, s	17.5, CH_3_	1.72, br s

^a 1^H at 500 MHz, ^13^C at 125 MHz. ^b^ Assignments aided by COSY, HSQC, HMBC, and NOESY experiments. ^c^ Assignments marked with the same letter in the same column may be interchanged.

**Table 5:** NMR data of diterpenoid **16** in CD_3_OD ^a,b^.

Position	δ_C_, Type	δ_H_, m (*J* in Hz)	Position	δ_C_, Type	δ_H_, m (*J* in Hz)
1	35.7, CH	2.50, m	11	17.8, CH_3_	0.92, d (7.0)
2	32.2, CH_2_	2.02, m 1.34, m	12	21.2, CH_3_	1.19, s
3	34.4, CH_2_	1.75, m 1.44, m	13	150.0, C	
4	49.1, C		14	110.5, CH_2_	4.94, br s 4.85, ^c^
5	28.1, CH_2_	2.05, m 1.40, m	15	37.7, CH_2_	1.89, m 1.84, m
6	16.0, CH_2_	1.89, m 1.52, m	16	27.4, CH_2_	2.09, m 2.05, m
7	45.1, CH	2.29, ddd (8.8,8.3,2.3)	17	125.6, CH	5.08, br t (7.0)
8	45.5, CH	2.38, dd (11.1,8.3)	18	132.2, C	
9	55.3, CH	2.59, dd (11.1,6.4)	19	25.9, CH_3_	1.65, br s
10	94.6, C		20	18.1, CH_3_	1.59, br s

^a 1^H at 500 MHz. ^13^C at 125 MHz. ^b^ Assignments aided by COSY, HSQC, HMBC, and NOESY experiments. ^c^ Obscured by the solvent signal.

**Table 6:** Highest concentration (μM) of diterpenoids **1**, **3**, **5**, **6**, **8**, **10**, **11**, **12**, **14,** and **16** that does not affect cellular viability of Bv2 cells and RAW 264.7 cells.

	Compound									
	1	3	5	6	8	10	11	12	14	16
Non-cytotoxic dose for Bv.2 (μM)	10	25	10	10	0.1	0.5	0.5	0.1	1	10
Non-cytotoxic dose for RAW 264.7 (μM)	1	25	10	10	0.1	0.5	0.5	1	10	10

## Supplementary Materials

The following supporting information can be downloaded at: https://www.mdpi.com/article/10.3390/md21040252/s1, Tables S1–S3: NMR data of compounds **7**, **9**–**12**, and **15**; Figures S1–S28: ^1^H and ^13^C NMR spectra of compounds **1**–**4** and **7**–**16**; Figure S29: Key COSY and HMBC correlations of compounds **2**–**4**, **7**, **9**–**12**, **14,** and **15**. Figure S30: Key NOESY correlations of compounds **2**–**4**, **7**, **9**–**12**, **14**, and **15**. Figure S31: Results of cytotoxicity assays on Bv.2 cells; Figure S32: Results of cytotoxicity assays on RAW 264.7 cells.
