# Supplementary material for: Rugulopteryx-Derived Spatane, Secospatane, Prenylcubebane and Prenylkelsoane Diterpenoids as Inhibitors of Nitric Oxide Production
Source: Mar Drugs. 2023 Apr 19;21(4):252. doi: 10.3390/md21040252 (PMC10142882; doi:10.3390/md21040252)
Supplement: Supplementary file 1 [file marinedrugs-21-00252-s001.zip › marinedrugs-2341513-supplementary.pdf]

Supplementary material

Rugulopteryx-Derived Spatane, Secospatane, Prenylcubebane and Prenylkelsoane Diterpenoids as Inhibitors of Nitric Oxide Production

Belén Cuevas [1,2], Ana I. Arroba [2,3], Carolina de los Reyes [1] and Eva Zubía [1,*]

[1] Departamento de Química Orgánica, Facultad de Ciencias del Mar y Ambientales, Universidad de Cádiz, 11510 Cádiz, Spain; belen.cuevas@inibica.es (B.C.); carolina.dereyes@uca.es (C.d.l.R.)

[2] Unidad de Investigación, Instituto de Investigación e Innovación Biomédica de Cádiz (INiBICA), Hospital Universitario Puerta del Mar, Avda. Ana de Viya 21, 11009 Cádiz, Spain; anaarroba@gmail.com

[3] Departamento de Biomedicina, Biotecnología y Salud Pública, Facultad de Medicina, Universidad de Cádiz, Pl. Falla, 9, 11003 Cádiz, Spain

* Correspondence: eva.zubia@uca.es

Table S1. ^{1}H and ^{13}C NMR data of diterpenoid **7** in CD_3OD

Table S2. ^{1}H and ^{13}C NMR data of diterpenoids **9-12** in CD_3OD

Table S3. ^{1}H and ^{13}C NMR data of diterpenoid **15** in CD_3OD

Figure S1. ^{1}H-RMN spectrum (500 MHz, CD_3OD) of okaspatol A (**1**)

Figure S2. ^{13}C-RMN spectrum (125 MHz, CD_3OD) of okaspatol A (**1**)

Figure S3. ^{1}H-RMN spectrum (500 MHz, CD_3OD) of okaspatol B (**2**)

Figure S4. ^{13}C-RMN spectrum (125 MHz, CD_3OD) of okaspatol B (**2**)

Figure S5. ^{1}H-RMN spectrum (500 MHz, CD_3OD) of okaspatol C (**3**)

Figure S6. ^{13}C-RMN spectrum (125 MHz, CD_3OD) of okaspatol C (**3**)

Figure S7. ^{1}H-RMN spectrum (500 MHz, CD_3OD) of okaspatol D (**4**)

Figure S8. ^{13}C-RMN spectrum (125 MHz, CD_3OD) of okaspatol D (**4**)

Figure S9. ^{1}H-RMN spectrum (500 MHz, CD_3OD) of compound **7**

Figure S10. ^{13}C-RMN spectrum (125 MHz, CD_3OD) of compound **7**

Figure S11. ^{1}H-RMN spectrum (500 MHz, CD_3OD) of rugukamural D (**8**)

Figure S12. ^{13}C-RMN spectrum (125 MHz, CD_3OD) of rugukamural D (**8**)

Figure S13. ^{1}H-RMN spectrum (500 MHz, CD_3OD) of compound **9**

Figure S14. ^{13}C-RMN spectrum (125 MHz, CD_3OD) of compound **9**

Figure S15. ^{1}H-RMN spectrum (500 MHz, CD_3OD) of compound **10**

Figure S16. ^{13}C-RMN spectrum (125 MHz, CD_3OD) of compound **10**

Figure S17. ^{1}H-RMN spectrum (500 MHz, CD_3OD) of compound **11**

Figure S18. ^{13}C-RMN spectrum (125 MHz, CD_3OD) of compound **11**

Figure S19. ^{1}H-RMN spectrum (500 MHz, CD_3OD) of compound **12**

Figure S20. ^{13}C-RMN spectrum (125 MHz, CD_3OD) of compound **12**

Figure S21. ^{1}H-RMN spectrum (500 MHz, CD_3OD) of okacubol A (**13**)

Figure S22. ^{13}C-RMN spectrum (125 MHz, CD_3OD) of okacubol A (**13**)

Figure S23. ^{1}H-RMN spectrum (500 MHz, CD_3OD) of okacubol B (**14**)

Figure S24. ^{13}C-RMN spectrum (125 MHz, CD_3OD) of okacubol B (**14**)

Figure S25. ^{1}H-RMN spectrum (500 MHz, CD_3OD) of compound **15**

Figure S26. ^{13}C-RMN spectrum (125 MHz, CD_3OD) of compound **15**

Figure S27. ^{1}H-RMN spectrum (500 MHz, CD_3OD) of okamurol A (**16**)

Figure S28. ^{13}C-RMN spectrum (125 MHz, CD_3OD) of okamurol A (**16**)

Figure S29. Key COSY and HMBC correlations observed for compounds **2**-**4**, **7**, **9**-**12**, **14**, and **15**

Figure S30. Key NOESY correlations observed for compounds **2**-**4**, **7**, **9**-**12**, **14**, and **15**

Figure S31. Cell survival of Bv.2 cells

Figure S32. Cell survival of RAW 264.7 cells

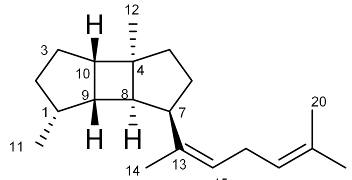

7

Table S1. NMR data of diterpenoid **7** in CD_3OD[a,b]

	**δ$_C$, Type**	**δ$_H$, m (*J* in Hz)**
1	38.1, CH	1.79, m
2	35.4, CH_2	1.70, m<br>1.29, dddd (12.2,12.2,12.2,6.3)
3	29.1, CH_2	1.70, m<br>1.46, m
4	42.9, C	
5	43.1, CH_2	1.64, m<br>1.43, m
6	29.8, CH_2	2.17, m<br>1.70, m
7	45.8, CH	2.80, ddd (12.8,5.8,5.8)
8	48.2, CH	1.96, br d (5.8, 4.7)
9	41.3, CH	2.29, ddd (7.0,7.0,4.7)
10	47.0, CH	2.11, m
11	14.2, CH_3	0.85, d (6.7)
12	20.8, CH_3	0.93, s
13	136.3, C	
14	24.4, CH_3	1.77, br s
15	126.2, CH	5.13, br t (7.2)
16	27.8, CH_2	2.67, m
17	124.8, CH	5.06, br t (7.2)
18	131.6, C	
19	25.9, CH_3	1.66, br s
20	17.8, CH_3	1.61, br s

[a] ^1H at 500 MHz, ^{13}C at 125 MHz; [b] Assignments aided by COSY, HSQC, HMBC, and NOESY experiments.

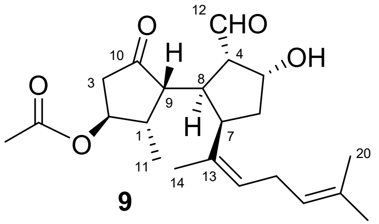
9

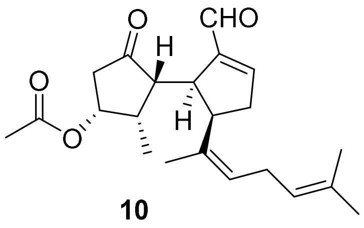
10

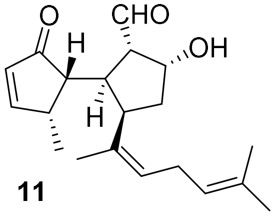
11

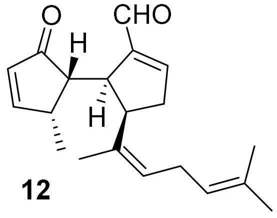
12

Table S2. NMR data of diterpenoids **9-12** in CD_3OD[a,b].

Position	9		10		11		12	
	δ_C, Type	δ_H, m (*J* in Hz)	δ_C, Type	δ_H, m (*J* in Hz)	δ_C, Type	δ_H, m (*J* in Hz)	δ_C, Type	δ_H, m (*J* in Hz)
1	41.1, CH	2.63, m	41.7, CH	2.57, m	41.8, CH	3.07, m	42.1, CH	3.07, m
2	76.8, CH	5.03, br d (6.2)	77.5, CH	5.07, m	170.7, CH	7.72, dd (5.8,3.0)	171.1, CH	7.66, dd (5.7,2.6)
3	42.2, CH_2	2.56, dd (19.6,6.2)<br>2.25, d (19.6)	43.0, CH_2	2.60, dd (19.1,6.2)<br>2.14, d (19.1)	132.4, CH	5.97, dd (5.8,1.6)	132.3, CH	5.90, dd (5.7,1.8)
4	61.3, CH	3.20, ddd (9.8,6.3,2.6)	149.9, C		60.5, CH	3.49, ddd (10.6,6.5,2.0)	149.7, C	
5	75.9, CH	4.77, ddd (6.3,6.1,3.4)	152.9, CH	7.01, br s	75.2, CH	4.82, [c]	155.4, CH	7.06, br s
6	40.8, CH_2	2.00, m<br>1.78, m	36.3, CH_2	2.75, m<br>2.57, m	40.5, CH_2	2.15, ddd (14.1,6.1,6.1)<br>1.79, ddd (14.1,8.5,3.0)	36.6, CH_2	2.89, dd (18.7,8.6)<br>2.55, ddd (18.7,8.6,3.1)
7	41.5, CH	3.61, m	46.3, CH	3.55, ddd (8.0,8.0,8.0)	41.9, CH	3.66, ddd (8.7,8.5,6.1)	46.3, CH	3.62, ddd (8.6,8.6,8.6)
8	38.2, CH	2.98, ddd (9.8,9.8,9.3)	41.9, CH	3.28, [c]	38.7, CH	3.05, m	42.9, CH	3.32, m
9	52.3, CH	2.55, m	49.8, CH	2.70, m	50.0, CH	2.31, dd (8.8,6.0)	48.7, CH	2.63, dd (6.2,3.4)
10	218.1, C		216.9, C		213.3, C		212.9, C	
11	13.9, CH_3	1.00, d (7.4)	14.0, CH_3	1.15, d (7.4)	17.6, CH_3	1.20, d (7.3)	16.0, CH_3	1.33, d (7.5)
12	204.7, CH	9.66, d (2.6)	191.5, CH	9.61, s	203.8, CH	9.65, d (2.0)	191.6, CH	9.55, s
13	136.2, C		135.4, C		136.2, C		135.7, C	
14	22.4, CH_3	1.64, br s	22.7, CH_3	1.74, br s	22.2, CH_3	1.64, d (1.3)	22.5, CH_3	1.79, br s
15	129.5, CH	5.22, br t (7.4)	130.0, CH	5.33, br t (7.2)	129.6, CH	5.27, br t (7.1)	129.7, CH	5.38, br t (7.3)
16	28.1, CH_2	2.79, m<br>2.72, m	28.0, CH_2	2.73, m	28.1, CH_2	2.82, m<br>2.75, m	28.1, CH_2	2.78, m<br>2.70, m
17	123.8, CH	5.06, br t (7.3)	123.8, CH	5.07, m	124.0, CH	5.07, br t (7.1)	123.8, CH	5.08, br t (7.0)
18	132.8, C		132.8, C		132.7, C		132.7, C	
19	25.9, CH_3	1.69, d (1.1)	25.9, CH_3	1.68, br s	25.9, CH_3	1.69, br s	25.9, CH_3	1.68, br s
20	17.9, CH_3	1.64, br s	17.9, CH_3	1.62, br s	17.9, CH_3	1.65, br s	17.9, CH_3	1.63, br s
CH_3*CO*O	172.0, C		172.1, C					
*CH_3*COO	21.0, CH_3	2.01, s	21.0, CH_3	2.01, s				

[a] ^1H at 500 MHz, ^{13}C at 125 MHz; [b] Assignments aided by COSY, HSQC, HMBC, and NOESY experiments; [c] Obscured by solvent signals

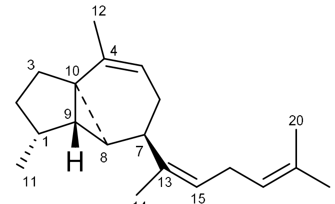

15

Table S3. NMR data of diterpenoid **15** in $CD_3OD^{a,b}$

Position	δC, Type	δH, m (*J* in Hz)
1	35.8, CH	2.29, m
2	30.8, CH_2	1.65, m<br>0.87, m
3	30.7, CH_2	2.09, ddd (12.1,11.7,8.3)<br>1.74, m
4	137.8, C	
5	118.2, CH	5.24. br d (6.9)
6	27.4, CH_2	1.78, m<br>1.62, m
7	33.5, CH	2.82, ddd (12.6,5.5,3.5)
8	24.6, CH	0.95, m
9	34.8, CH	1.62, m
10	31.9, C	
11	18.2, CH_3	1.00, d (6.6)
12	21.7, CH_3	1.82, br s
13	140.2, C	
14	20.3, CH_3	1.71, br s
15	124.66, CH	5.13, br t (7.3)
16	27.3, CH_2	2.70, m
17	124.74, CH	5.07, br t (7.2)
18	131.8, C	
19	25.9, CH_3	1.69, br d (1.1)
20	17.8, CH_3	1.61, br s

[a] ^{1}H at 500 MHz, ^{13}C at 125 MHz; [b] Assignments aided by COSY, HSQC, HMBC, and NOESY experiments.

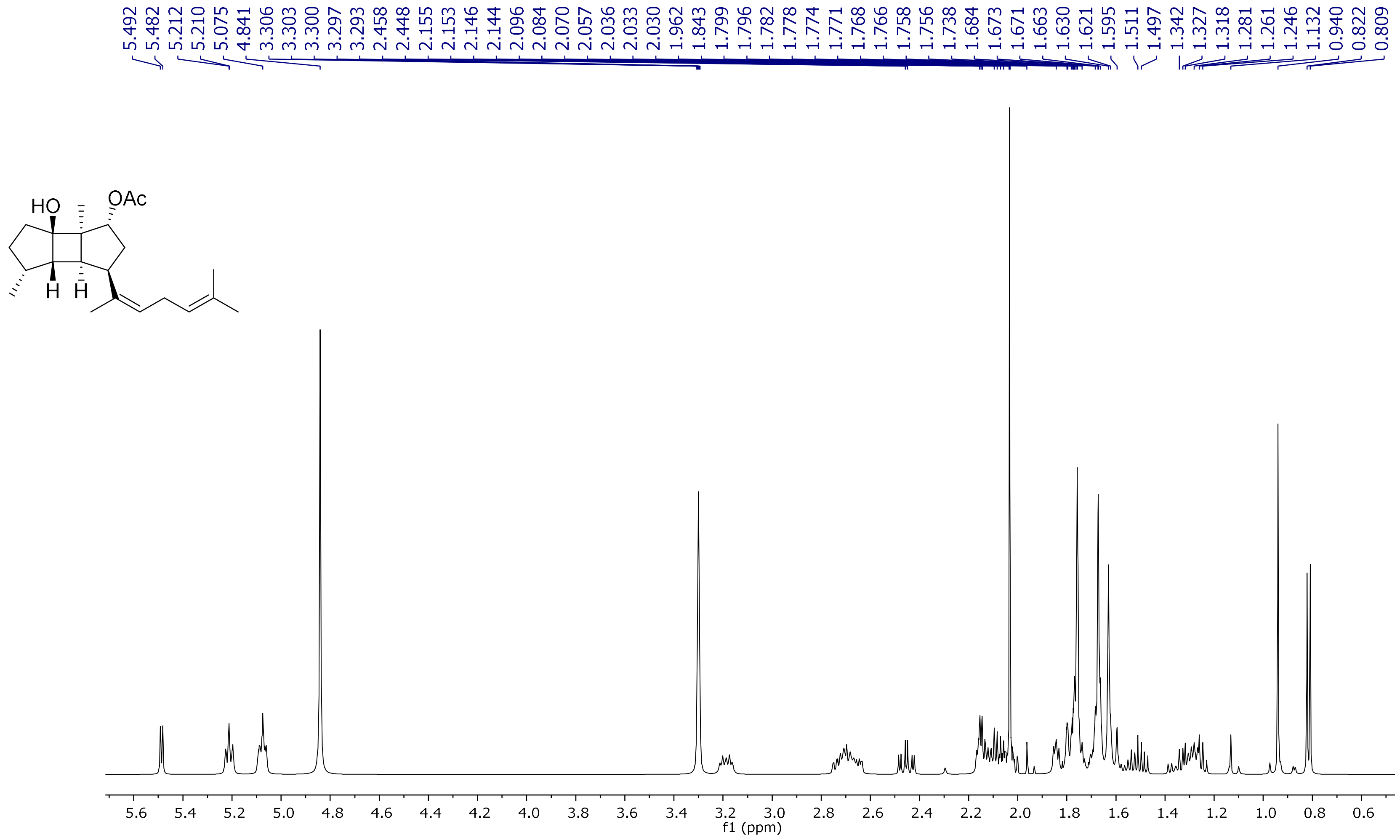

Figure S1. ^{1}H RMN spectrum (500 MHz, CD_3OD) of okaspatol A (**1**)

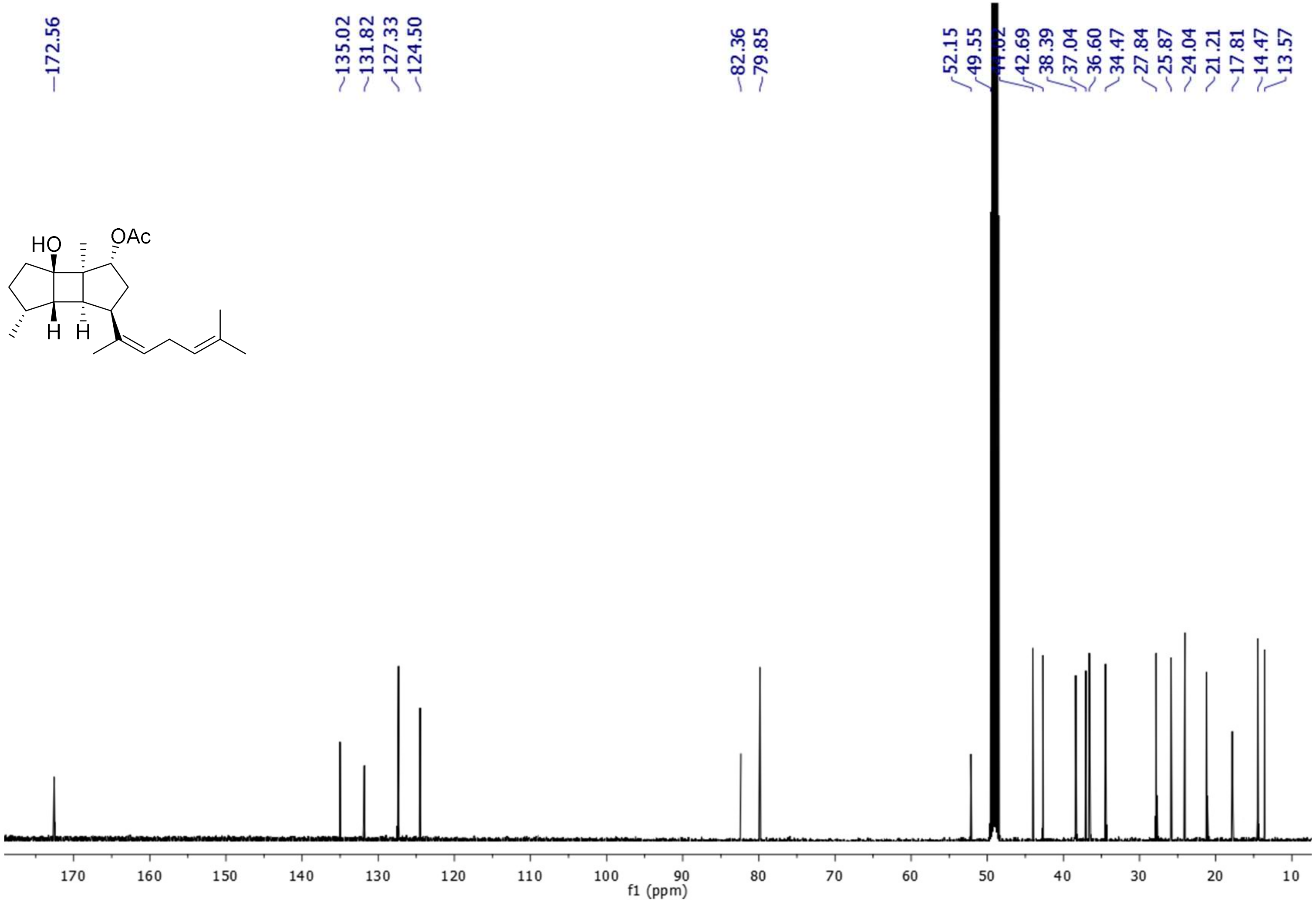

Figure S2. ^{13}C RMN spectrum (125 MHz, CD_3OD) of okaspatol A (**1**)

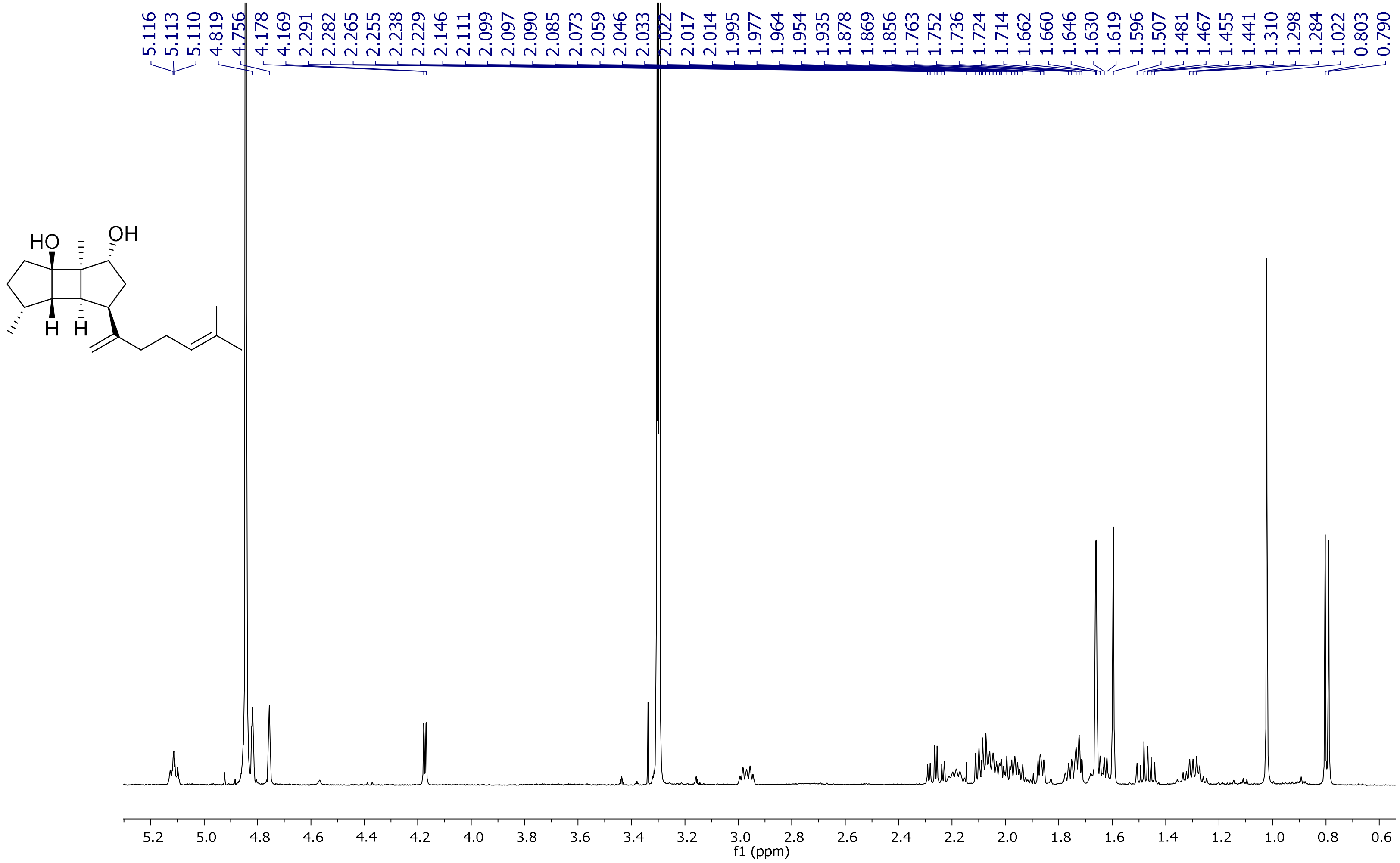

Figure S3. ^{1}H RMN spectrum (500 MHz, CD_3OD) of okaspatol B (**2**)

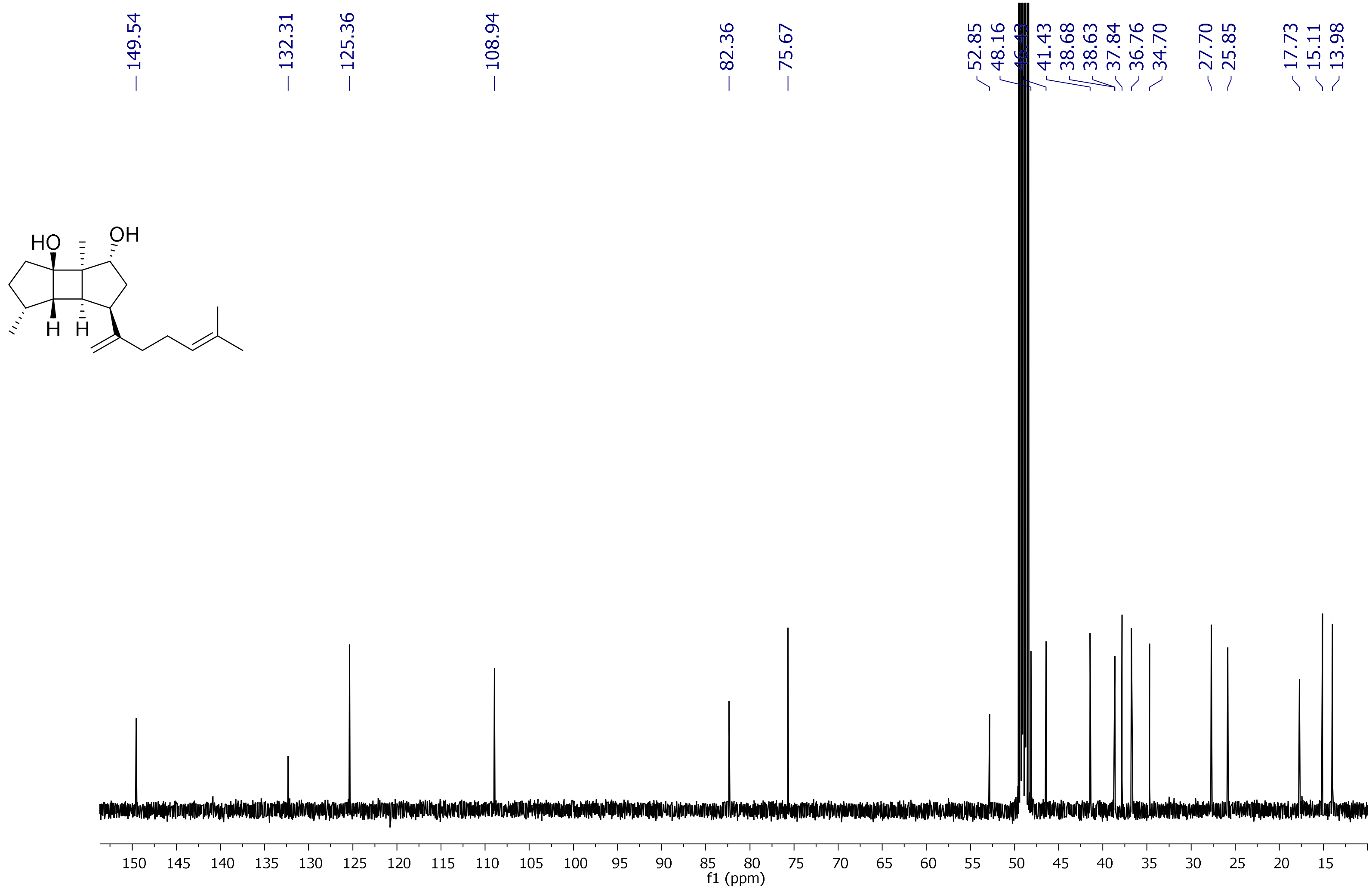

Figure S4. ^{13}C RMN spectrum (125 MHz, CD_3OD) of okaspatol B (**2**)

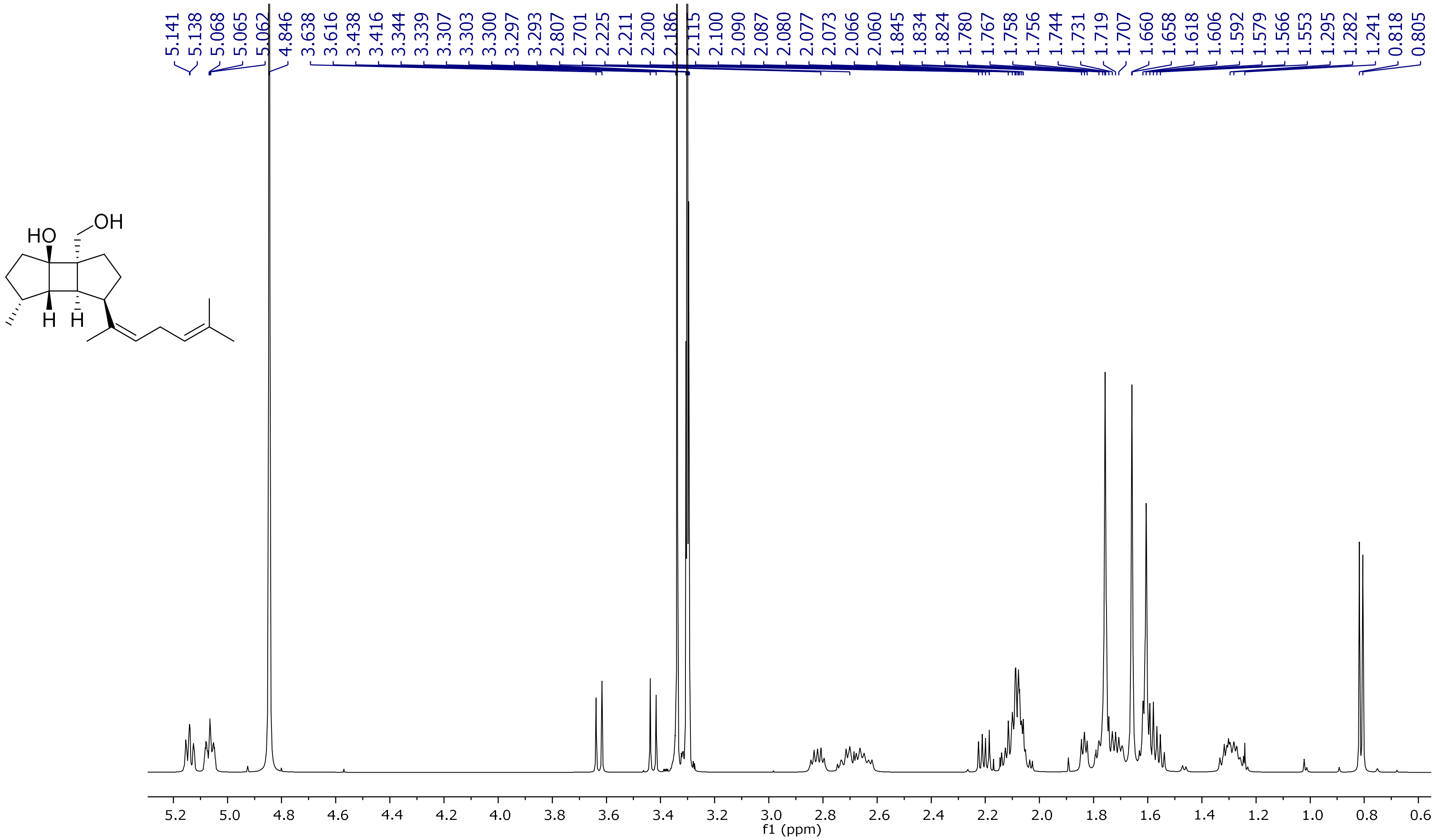

Figure S5. ^{1}H RMN spectrum (500 MHz, CD_3OD) of okaspatol C (**3**)

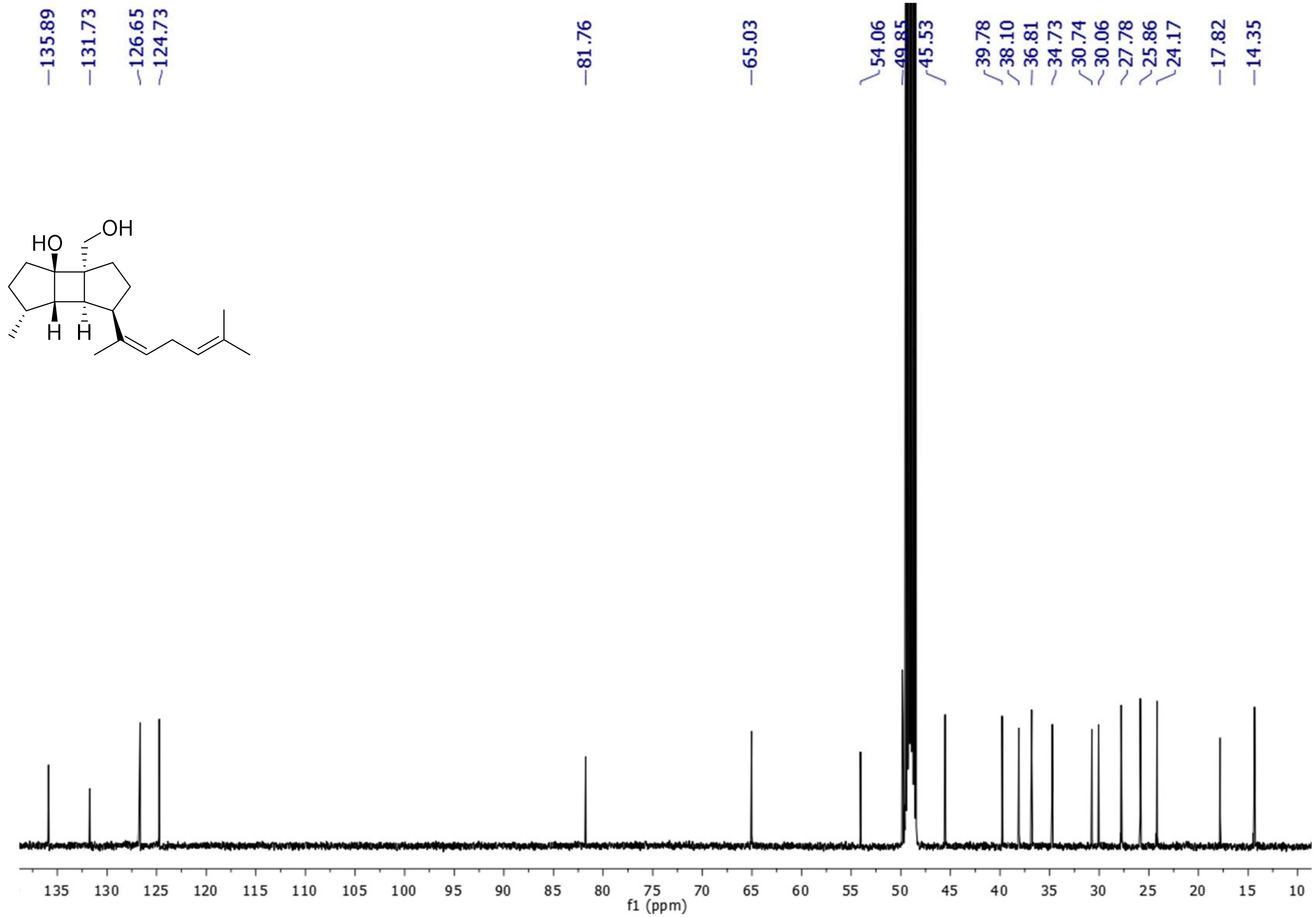

Figure S6. ^{13}C RMN spectrum (125 MHz, CD_3OD) of okaspatol C (**3**)

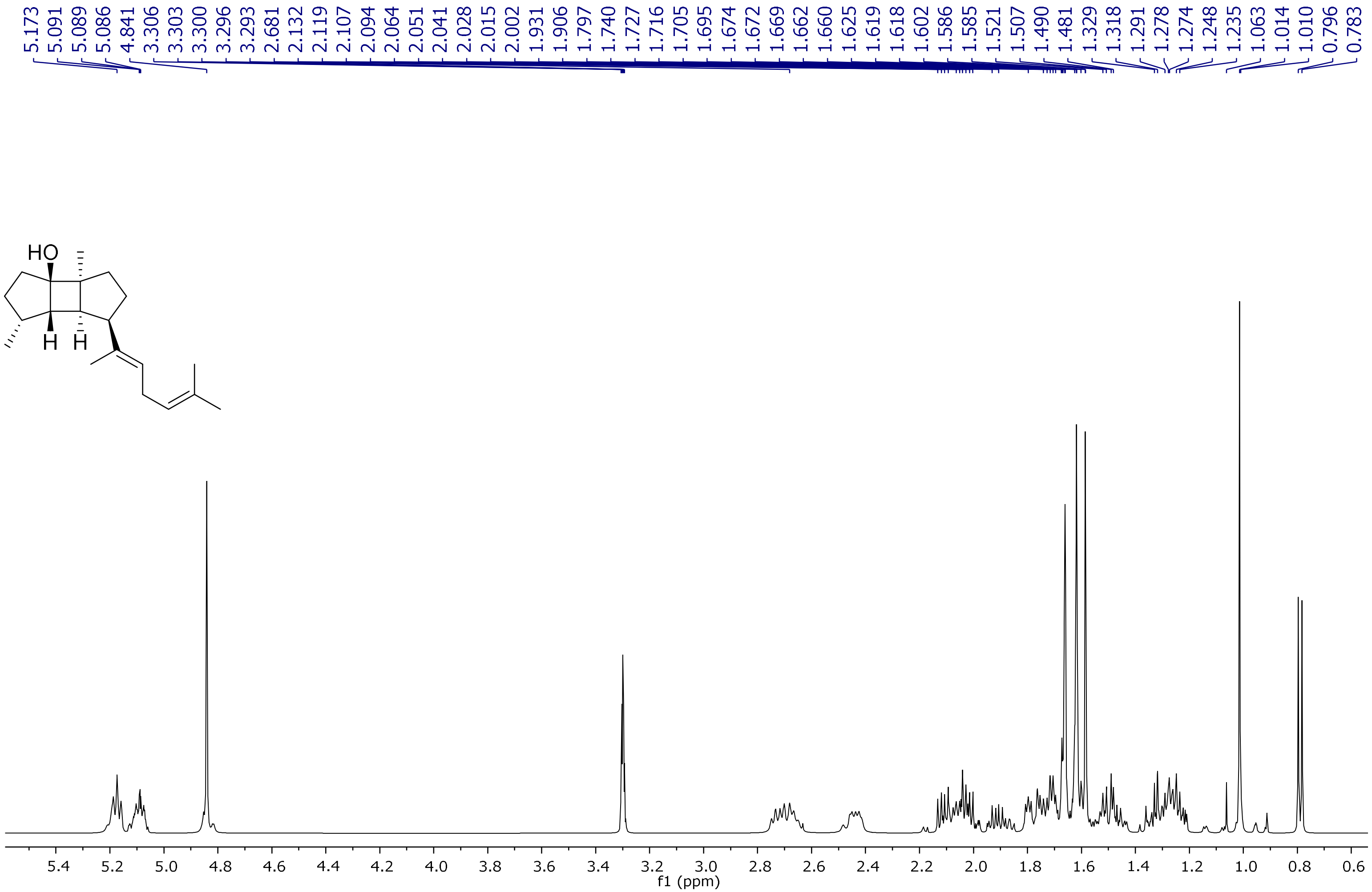

Figure S7. ^{1}H RMN spectrum (500 MHz, CD_3OD) of okaspatol D (**4**)

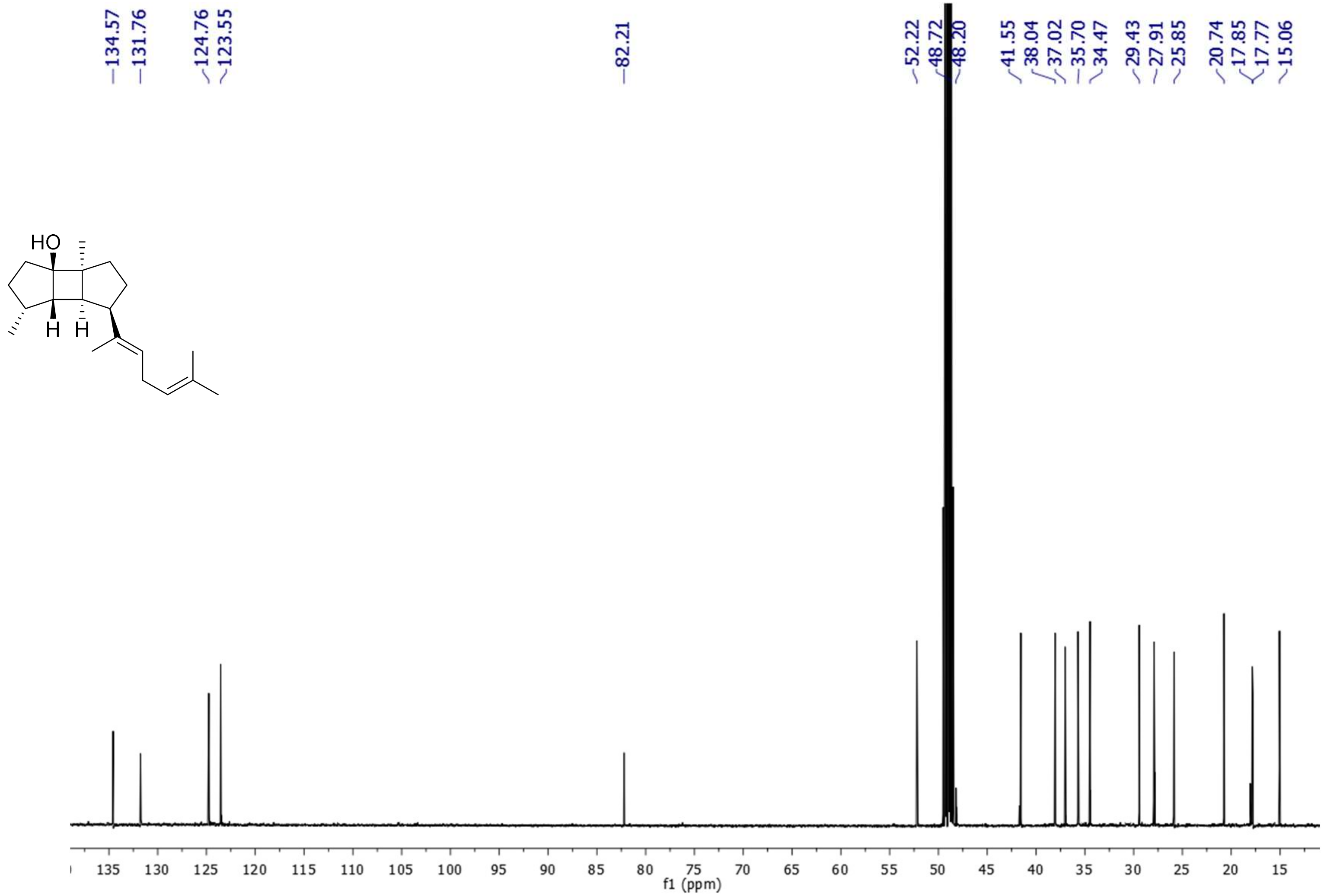

Figure S8. ^{13}C RMN spectrum (125 MHz, CD_3OD) of okaspatol D (**4**)

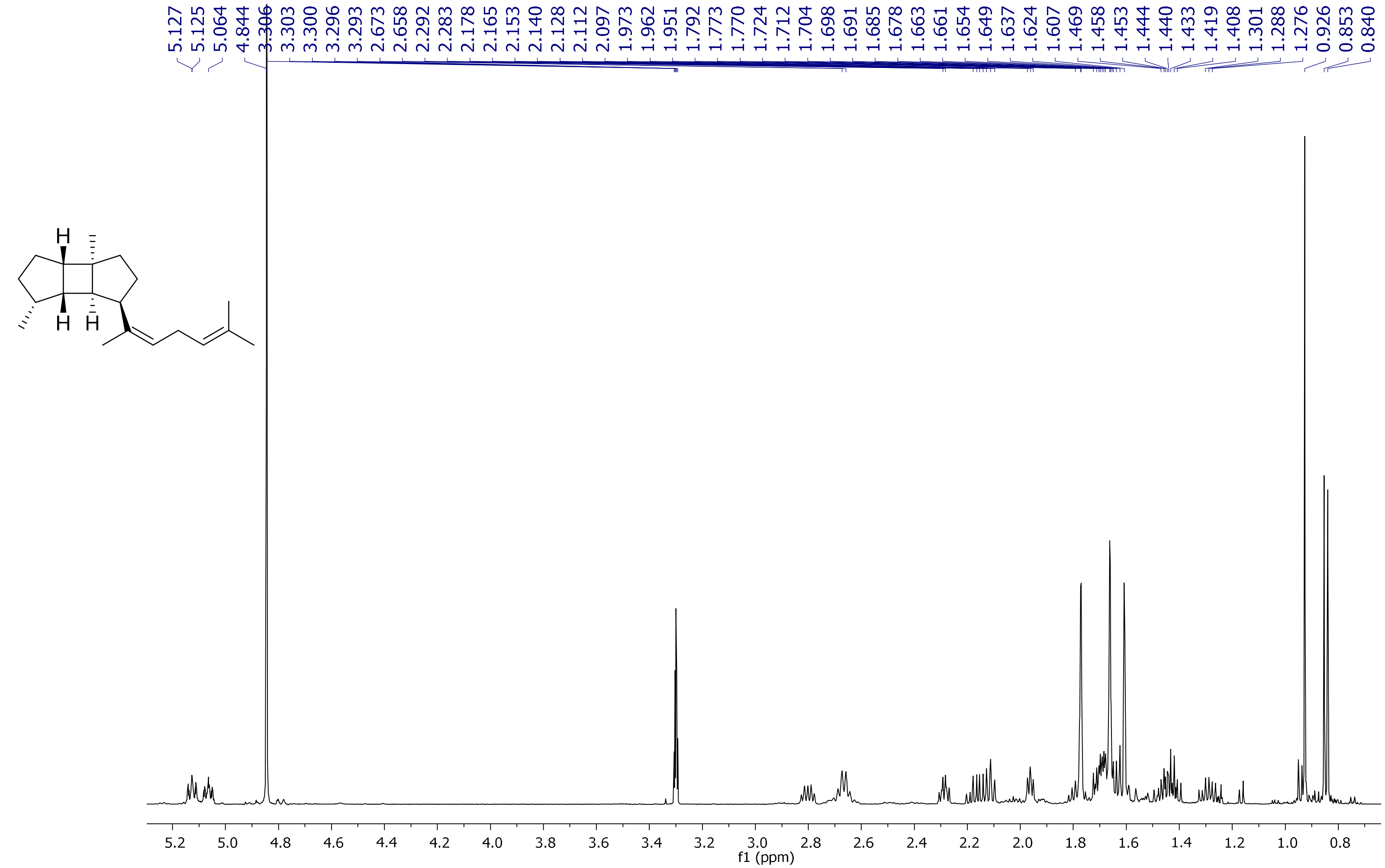

Figure S9. ^{1}H RMN spectrum (500 MHz, CD_3OD) of compound **7**

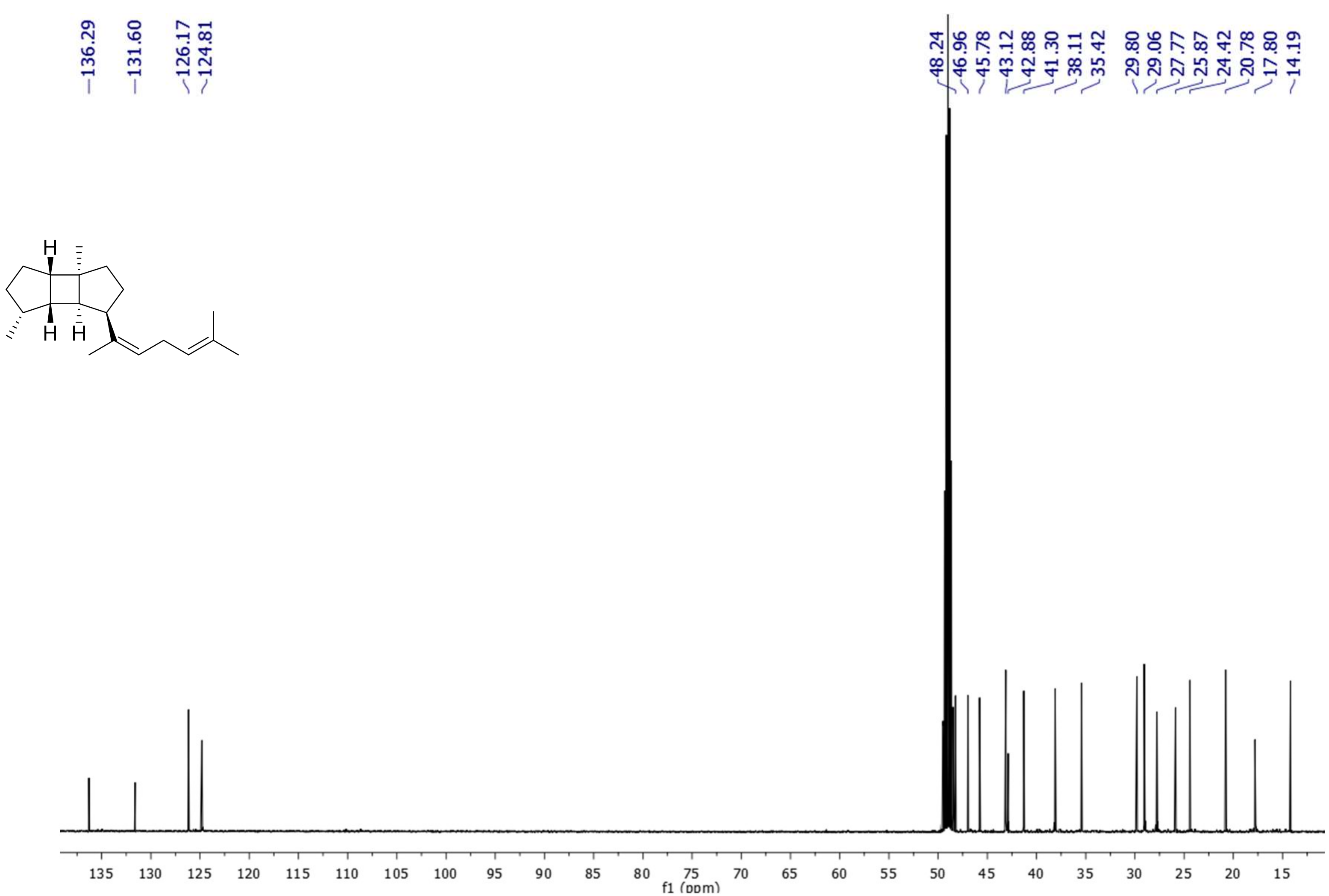

Figure S10. ^{13}C RMN spectrum (125 MHz, CD_3OD) of compound **7**

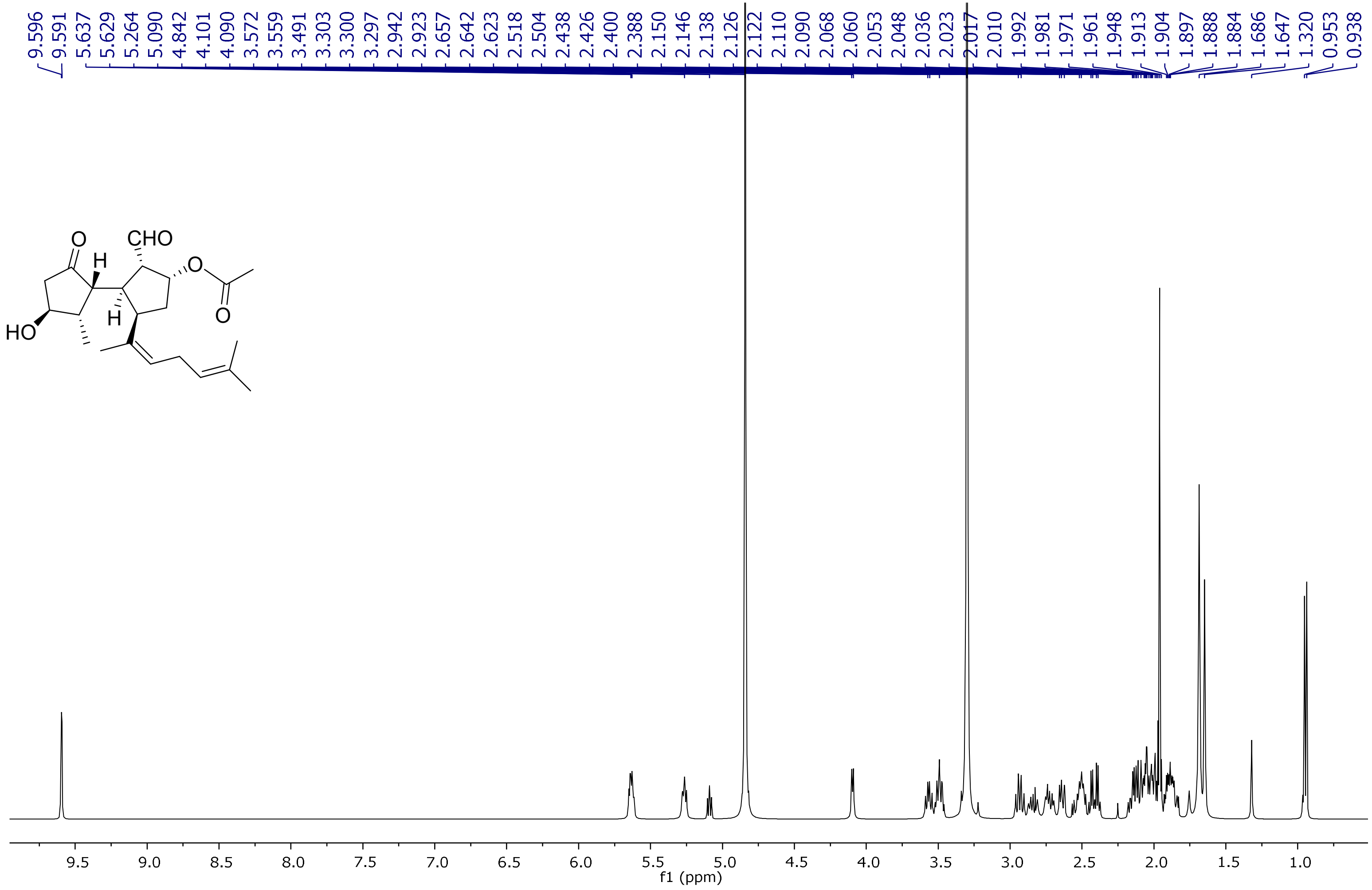

Figure S11. ^{1}HRMN spectrum (500 MHz, CD_3OD) of rugukamural D (**8**)

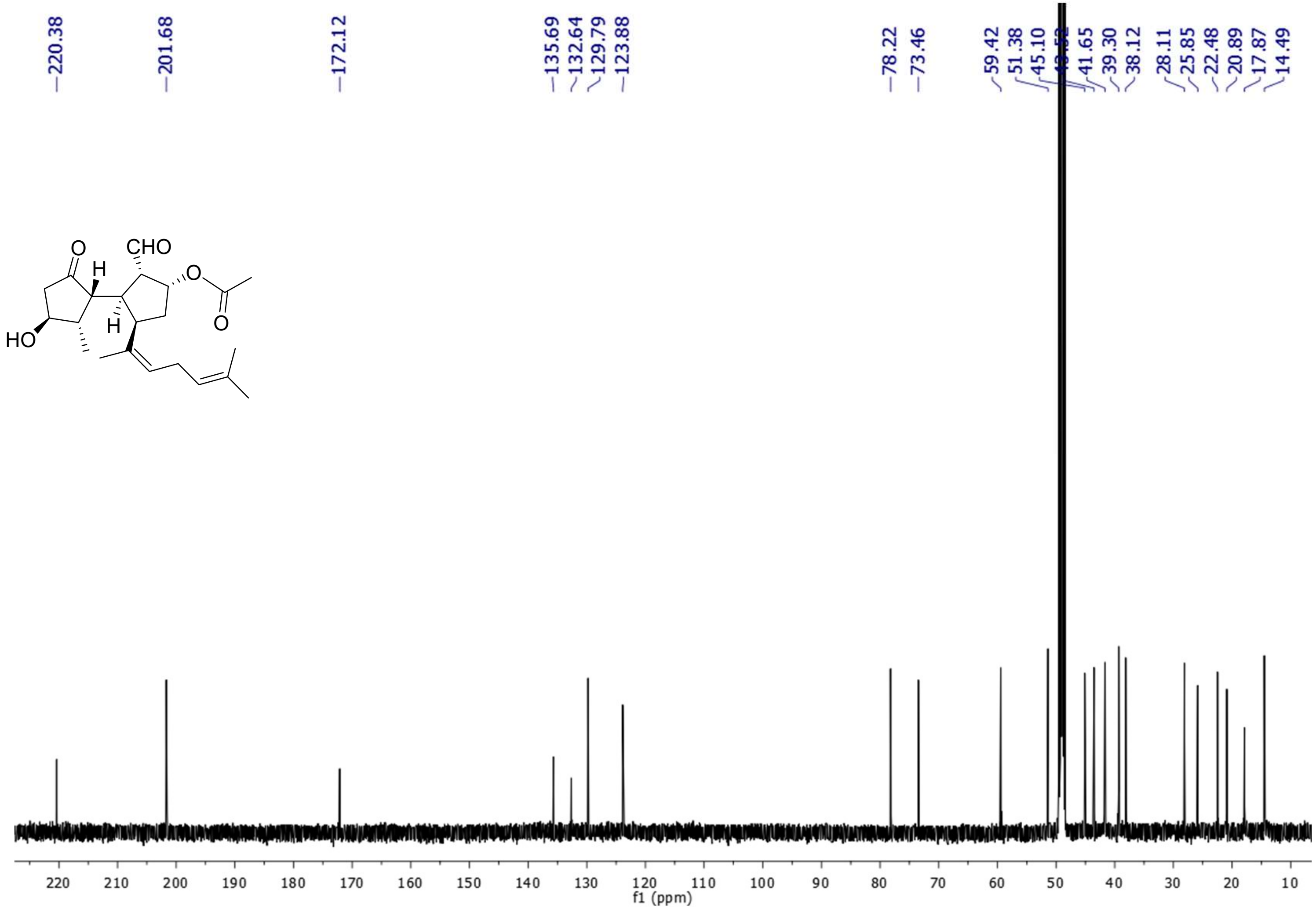

Figure S12. ^{13}C RMN spectrum (125 MHz, CD_3OD) of rugukamural D (**8**)

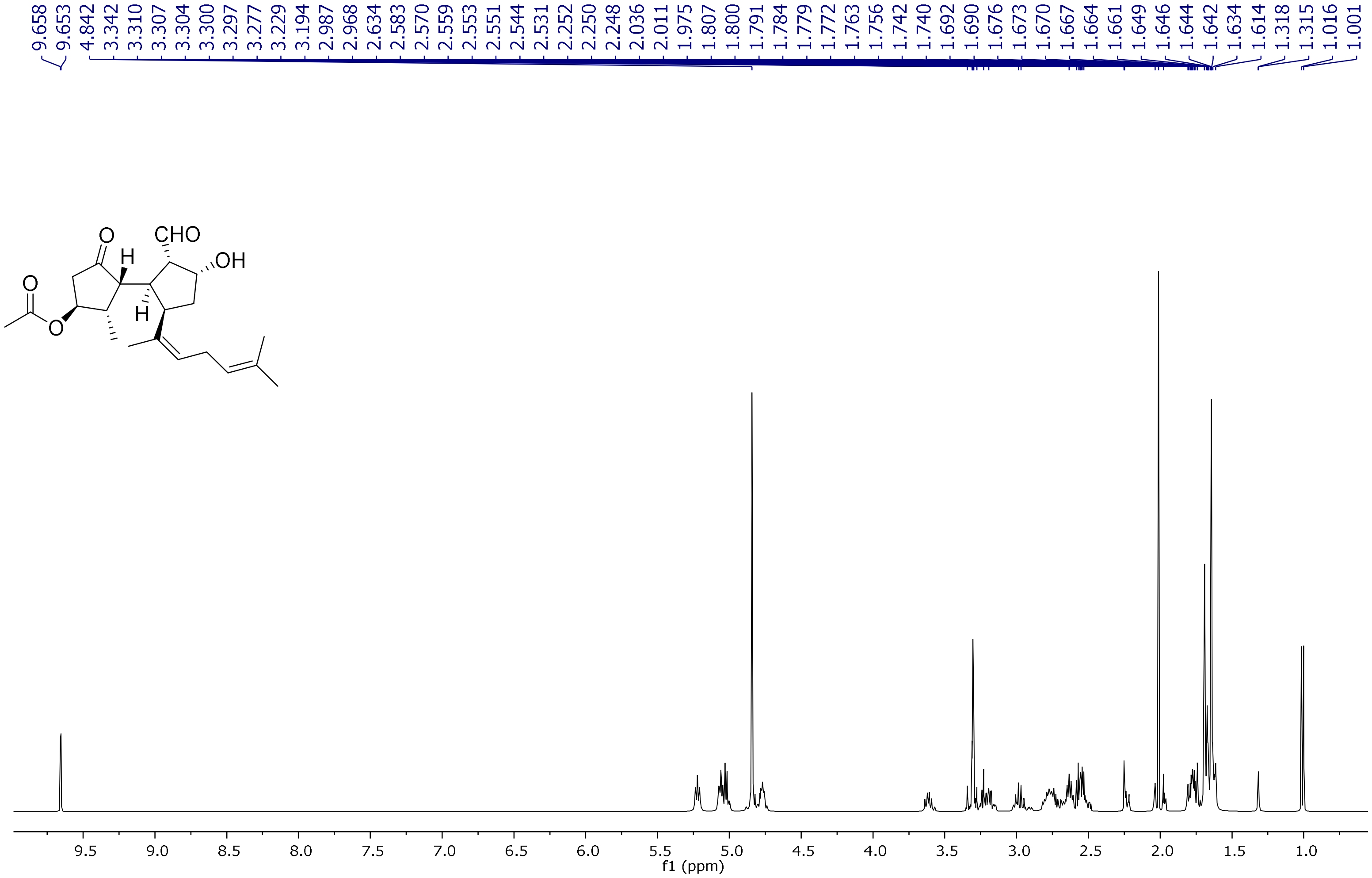

Figure S13. ^{1}H RMN spectrum (500 MHz, CD_3OD) of compound **9**

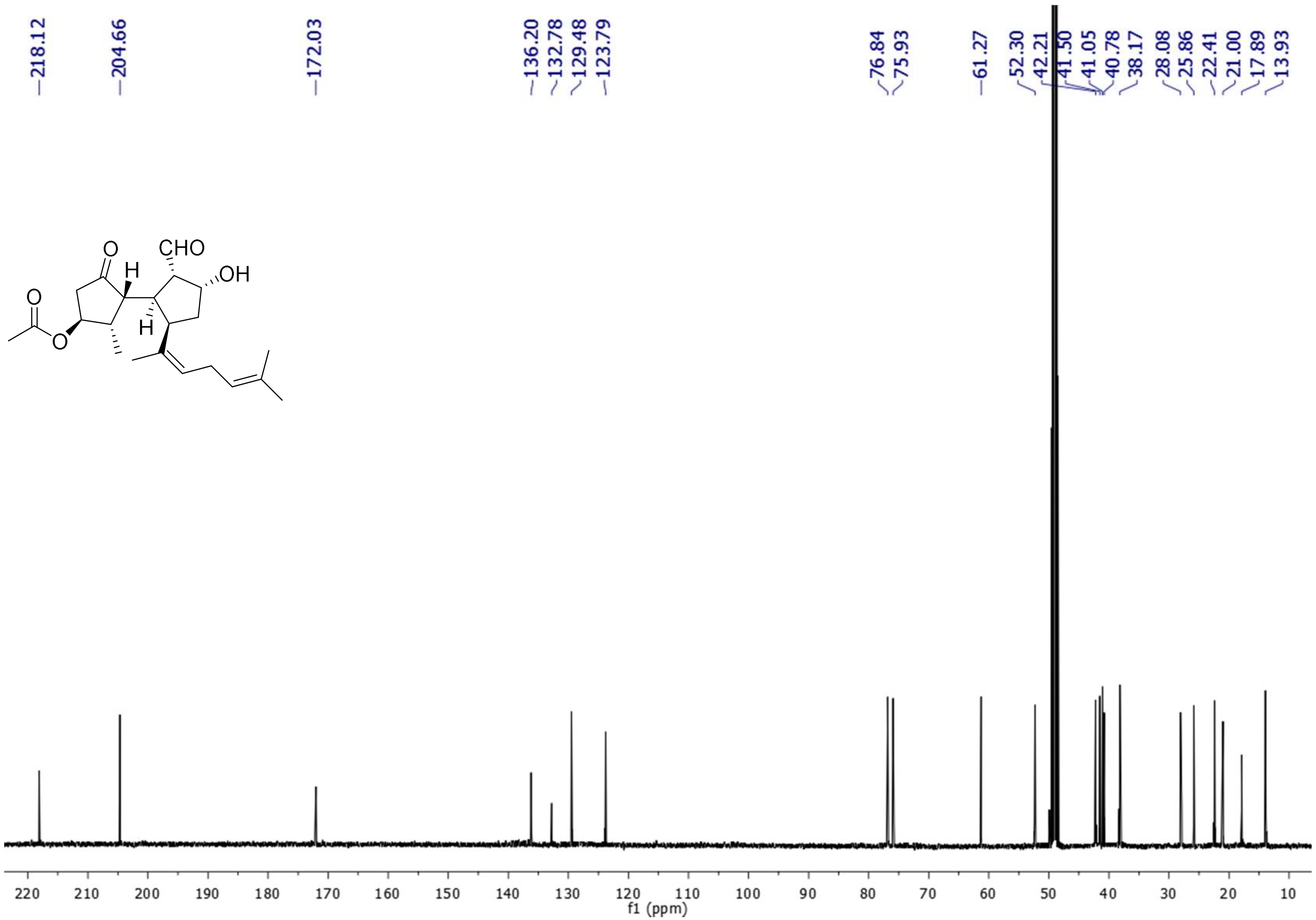

Figure S14. ^{13}C RMN spectrum (125 MHz, CD_3OD) of compound **9**

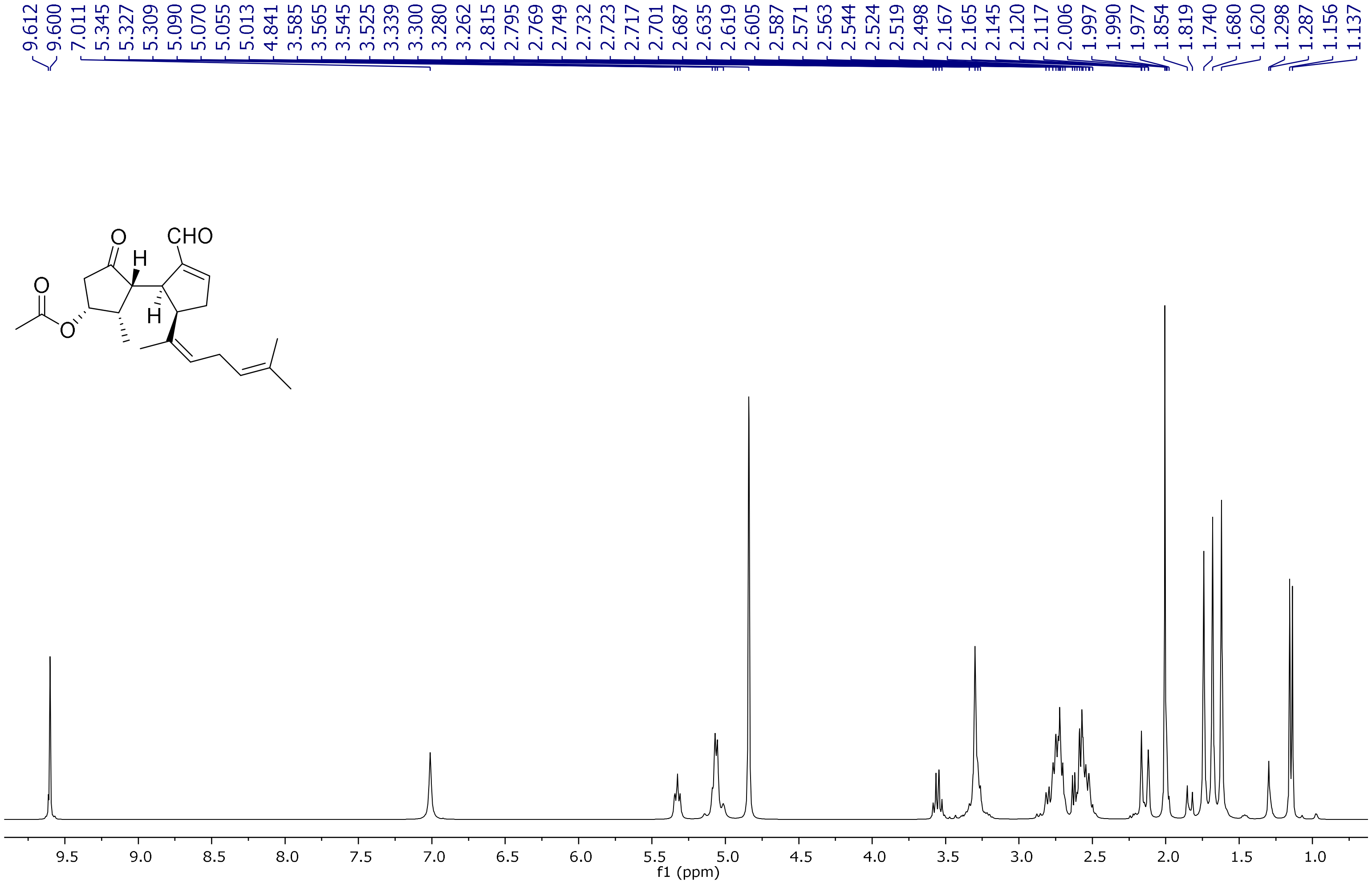

Figure S15. ^{1}H RMN spectrum (500 MHz, CD_3OD) of compound **10**

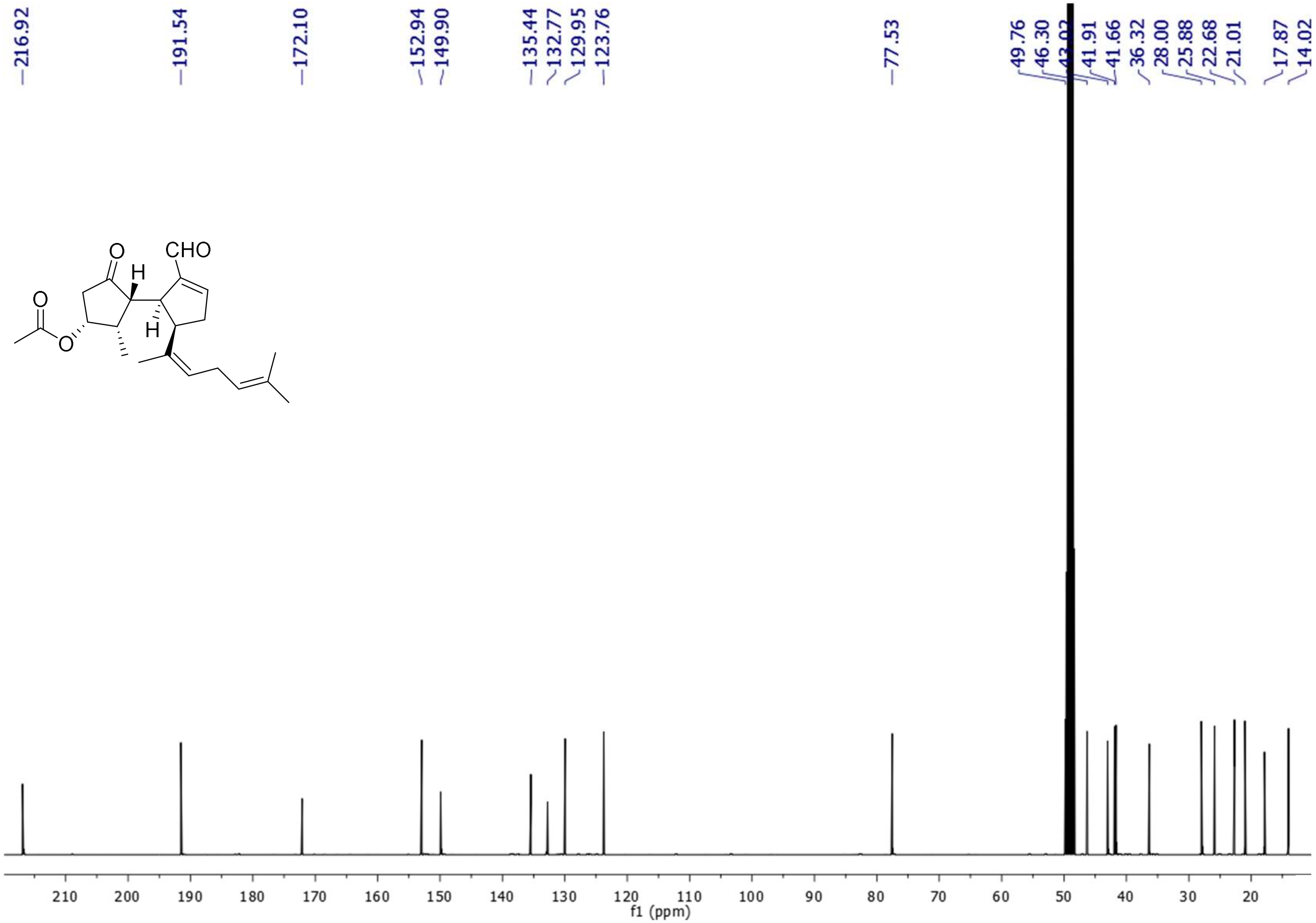

Figure S16. ^{13}C RMN spectrum (125 MHz, CD_3OD) of compound **10**

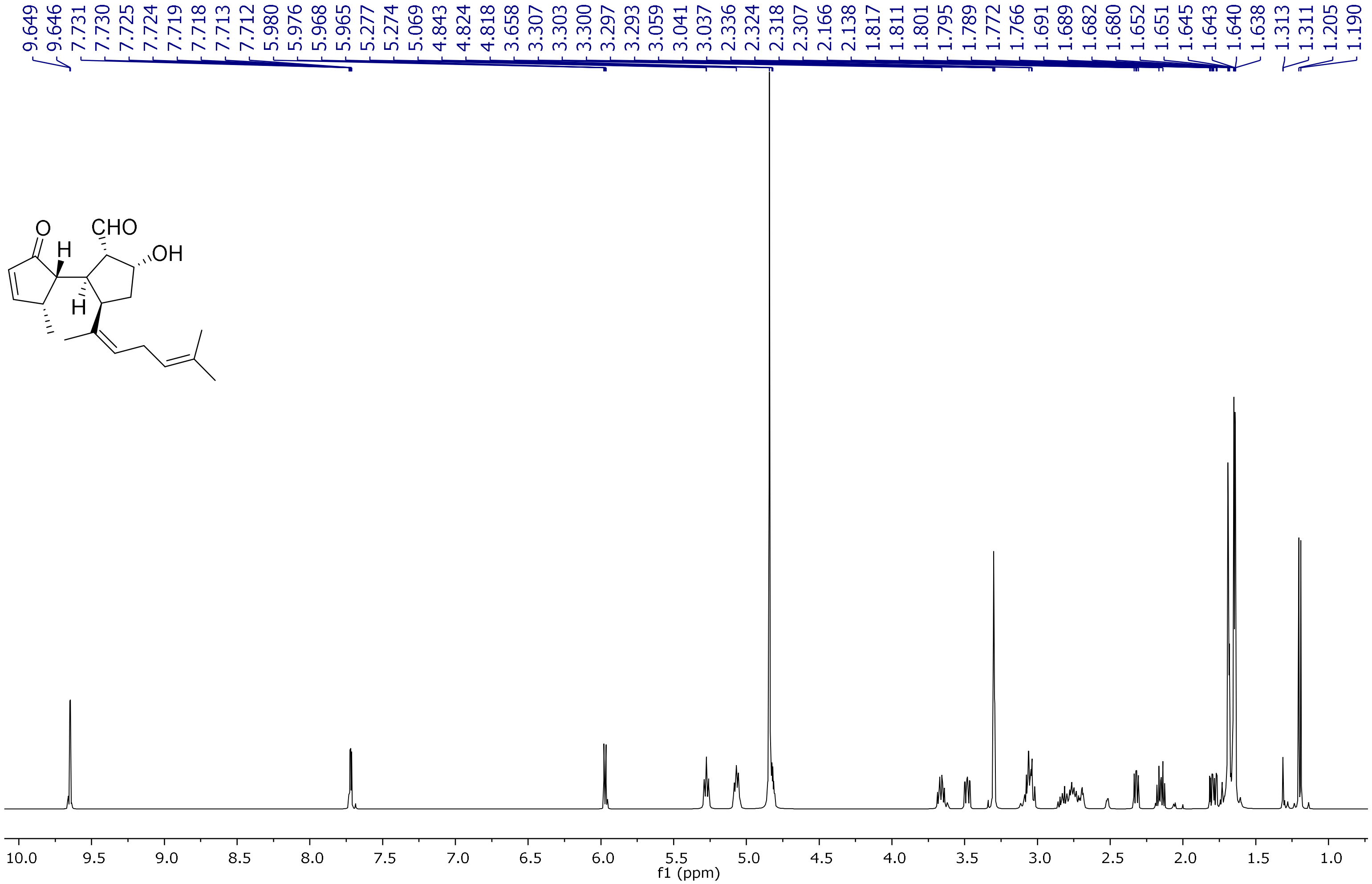

Figure S17. ^{1}H RMN spectrum (500 MHz, CD_3OD) of compound **11**

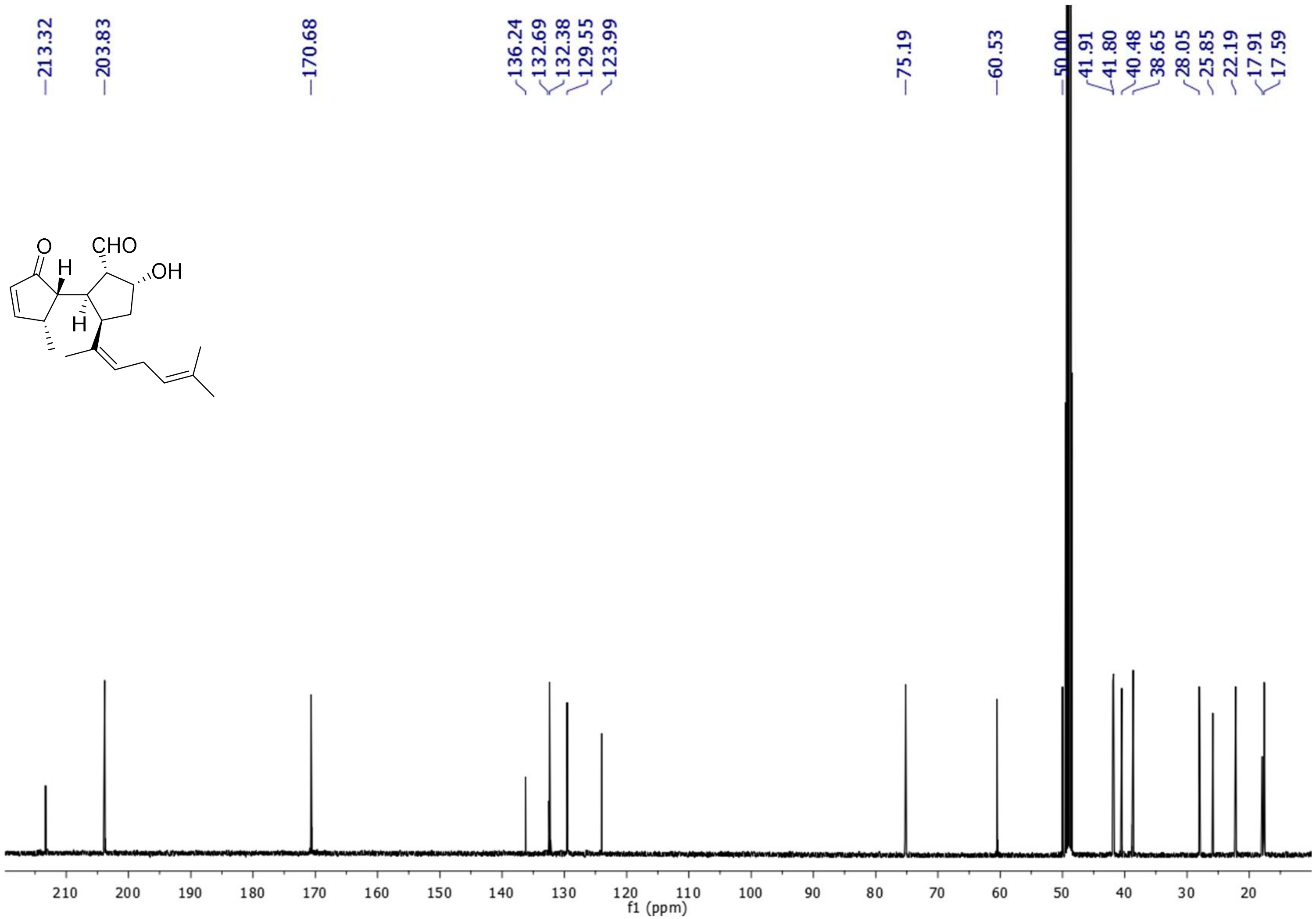

Figure S18. ^{13}C RMN spectrum (125 MHz, CD_3OD) of compound **11**

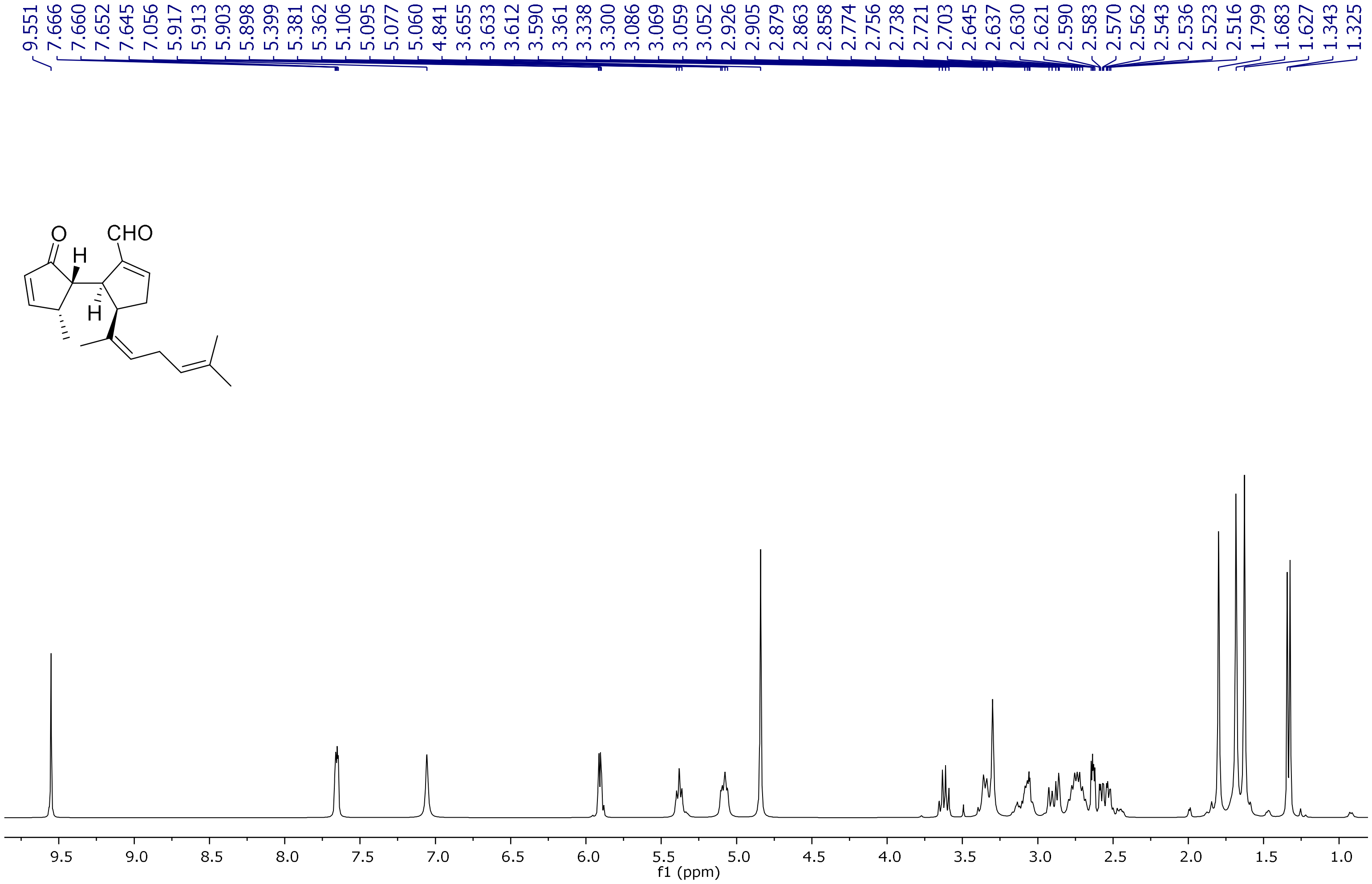

Figure S19. ^{1}H RMN spectrum (500 MHz, CD_3OD) of compound **12**

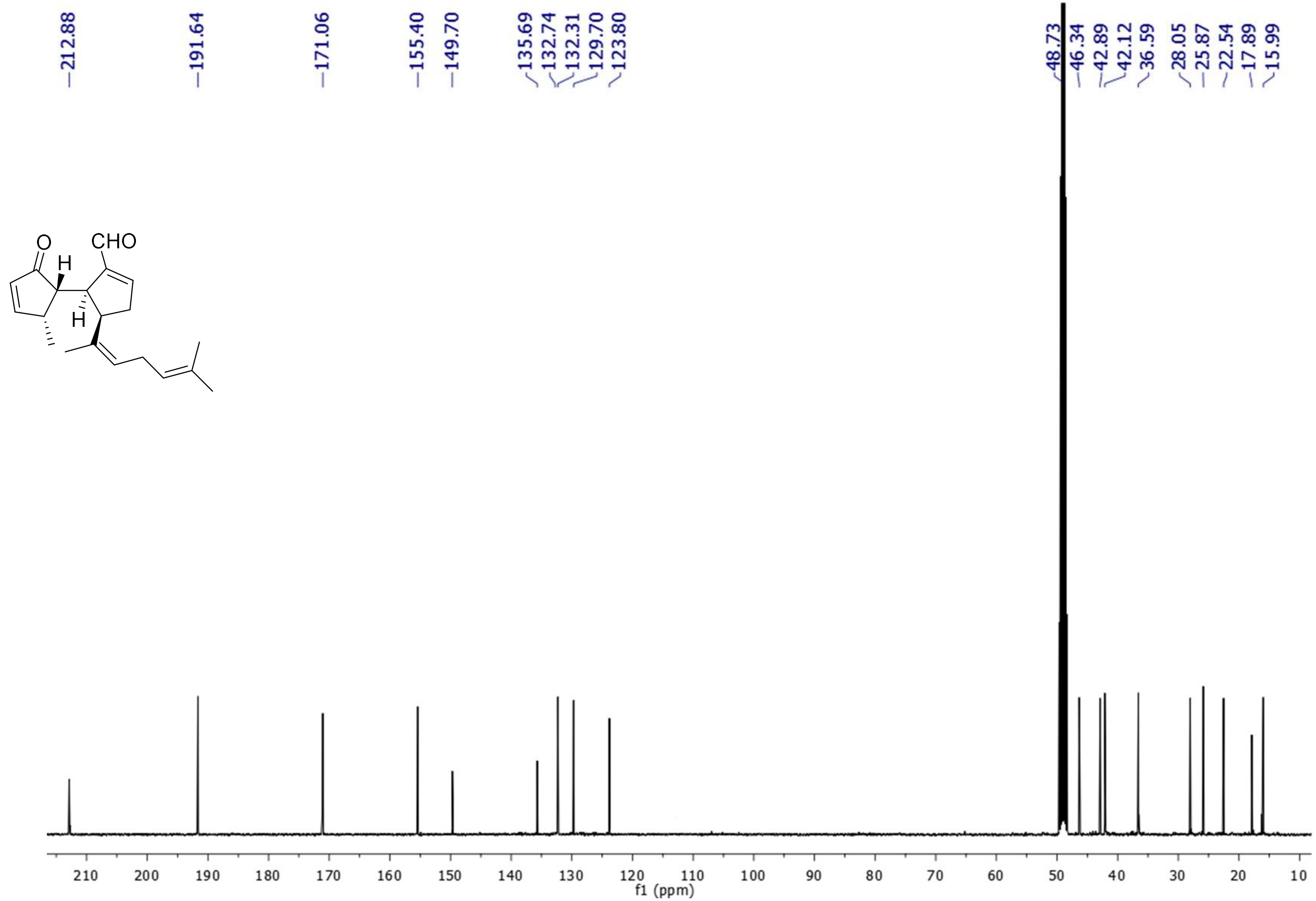

Figure S20. ^{13}C RMN spectrum (125 MHz, CD_3OD) of compound **12**

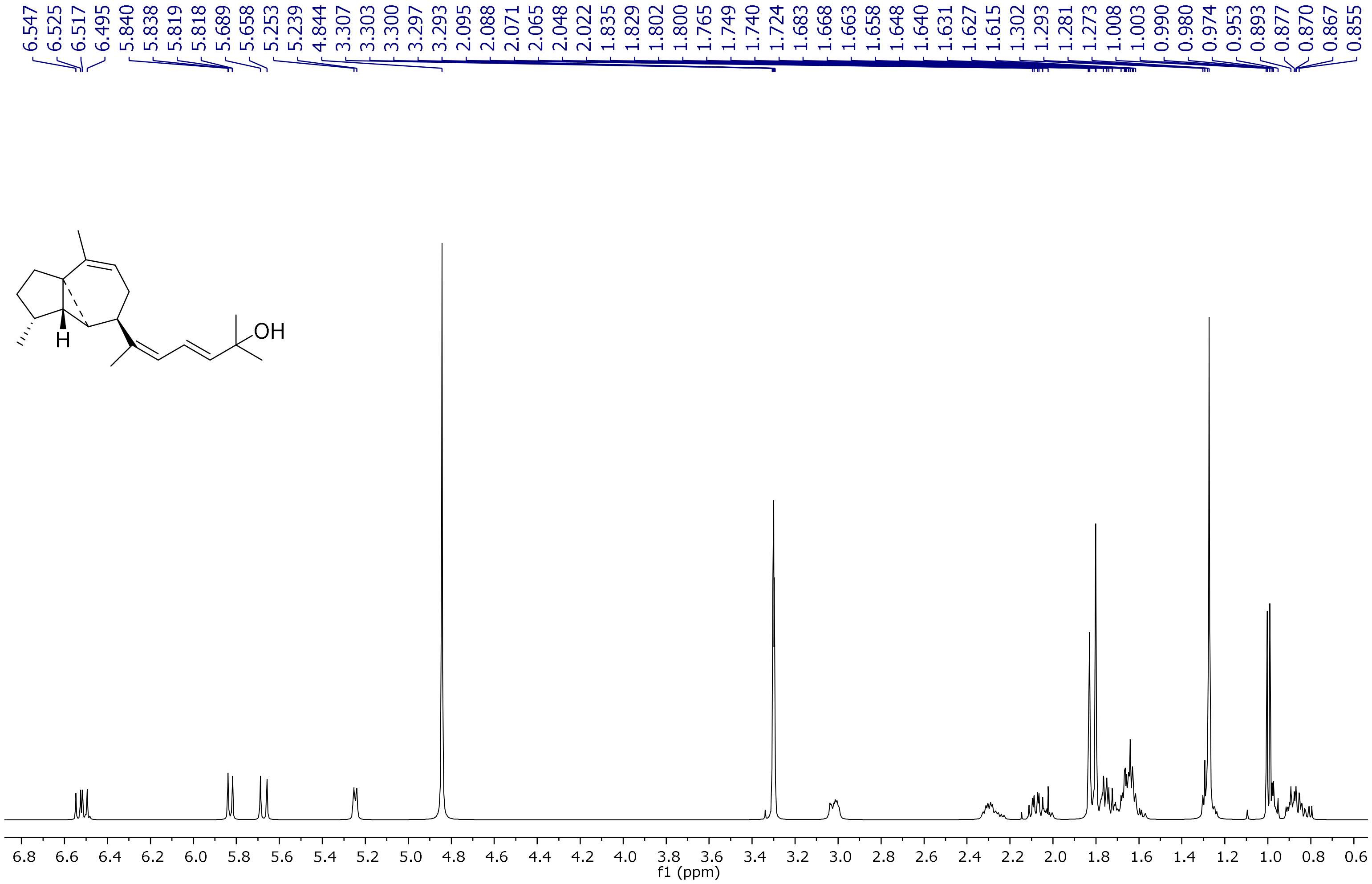

Figure S21. ^{1}H RMN spectrum (500 MHz, CD_3OD) of okacubol A (**13**)

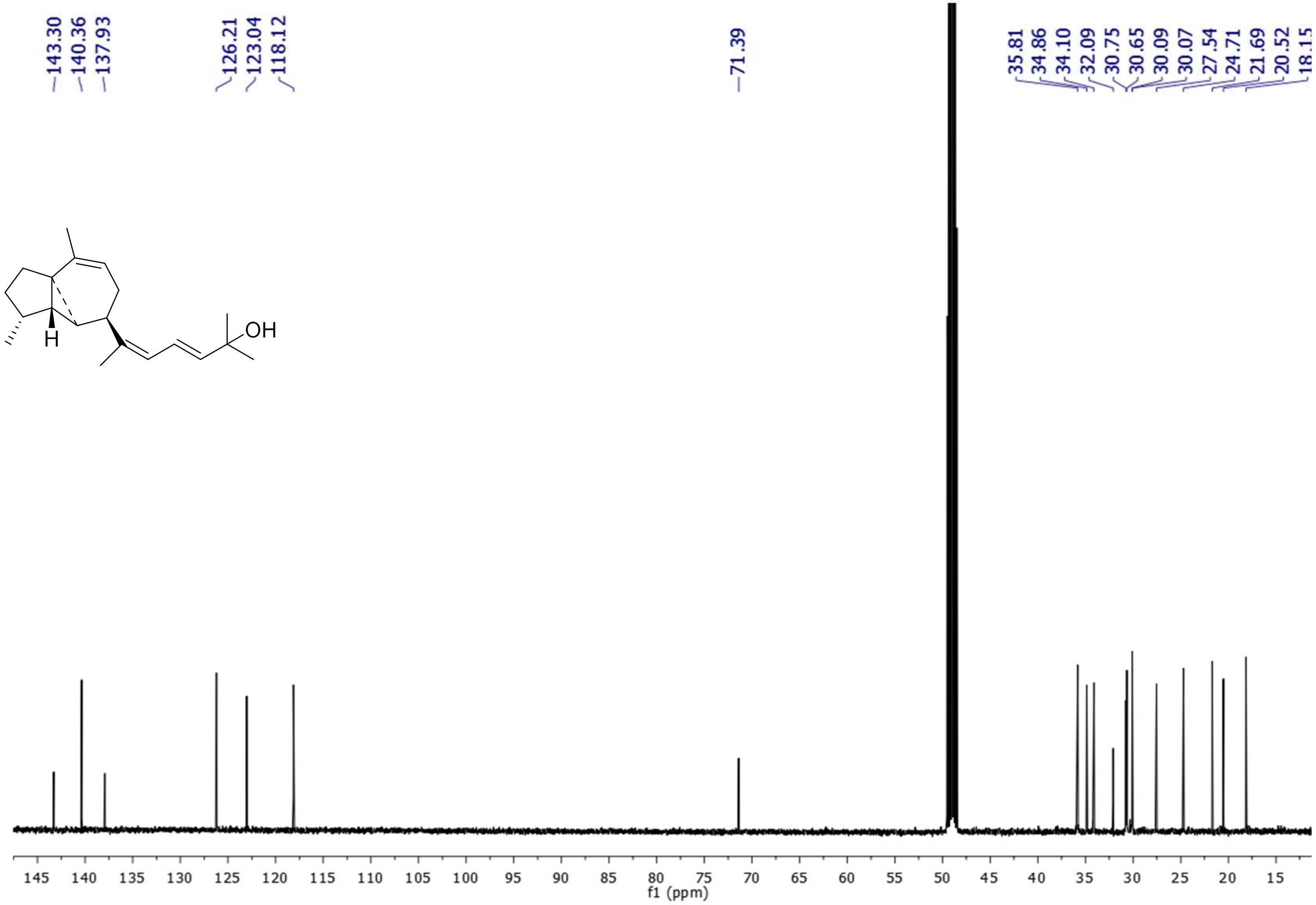

Figure S22. ^{13}C RMN spectrum (125 MHz, CD_3OD) of okacubol A (**13**)

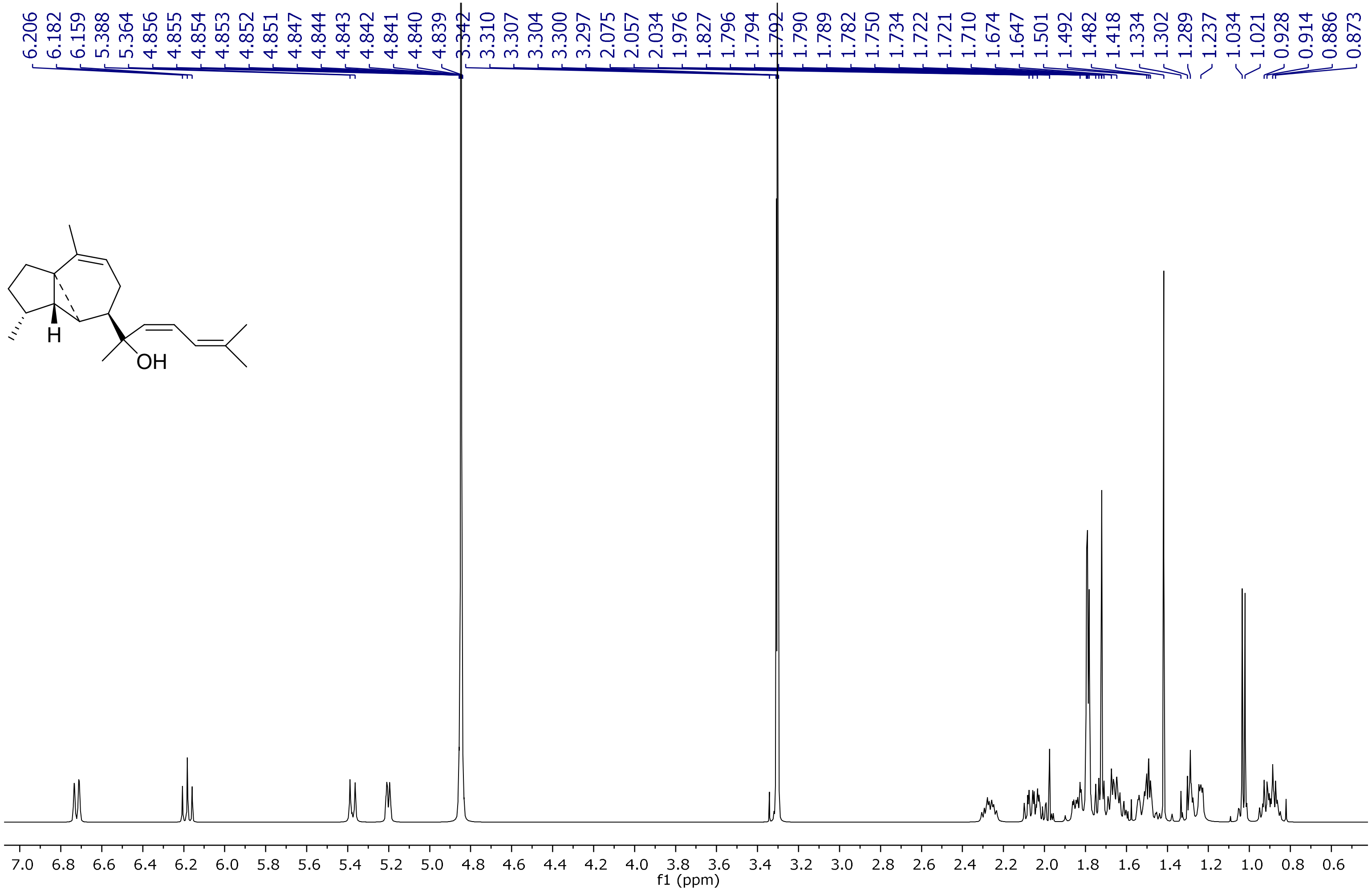

Figure S23. ^{1}H RMN spectrum (500 MHz, CD_3OD) of okacubol B (**14**)

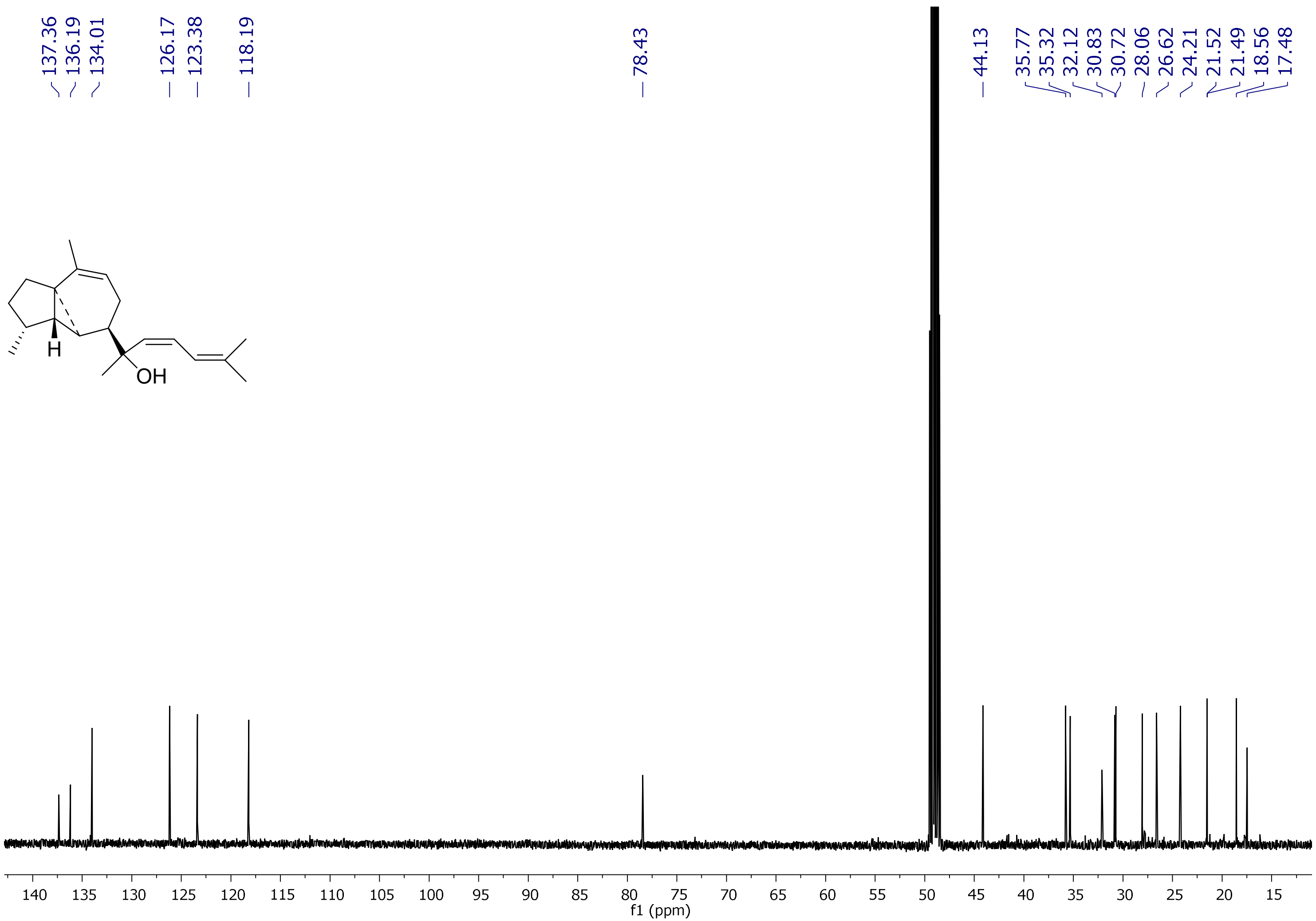

Figure S24. ^{13}C RMN spectrum (125 MHz, CD_3OD) of okacubol A (**14**)

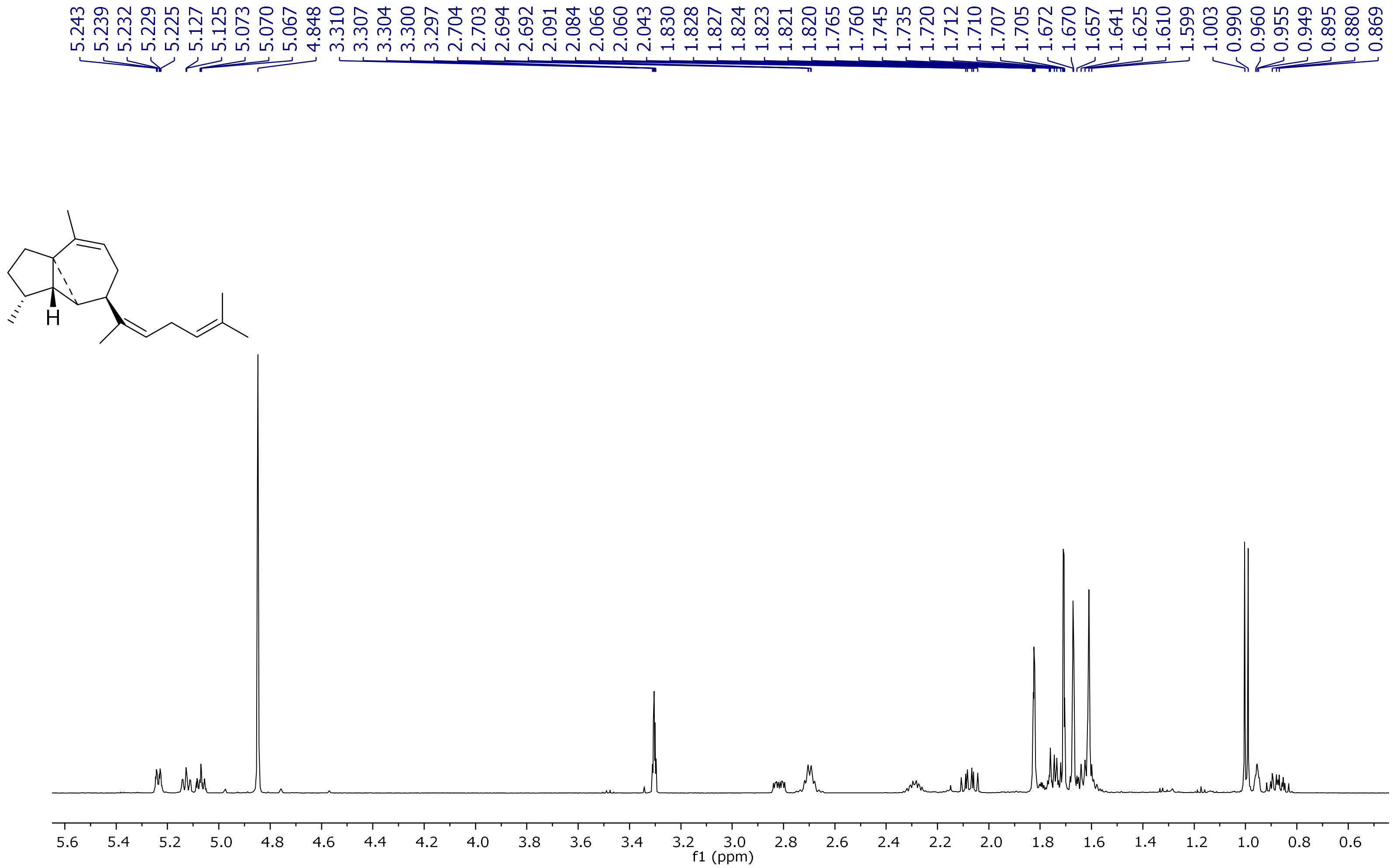

Figure S25. ^{1}H RMN spectrum (500 MHz, CD_3OD) of compound **15**

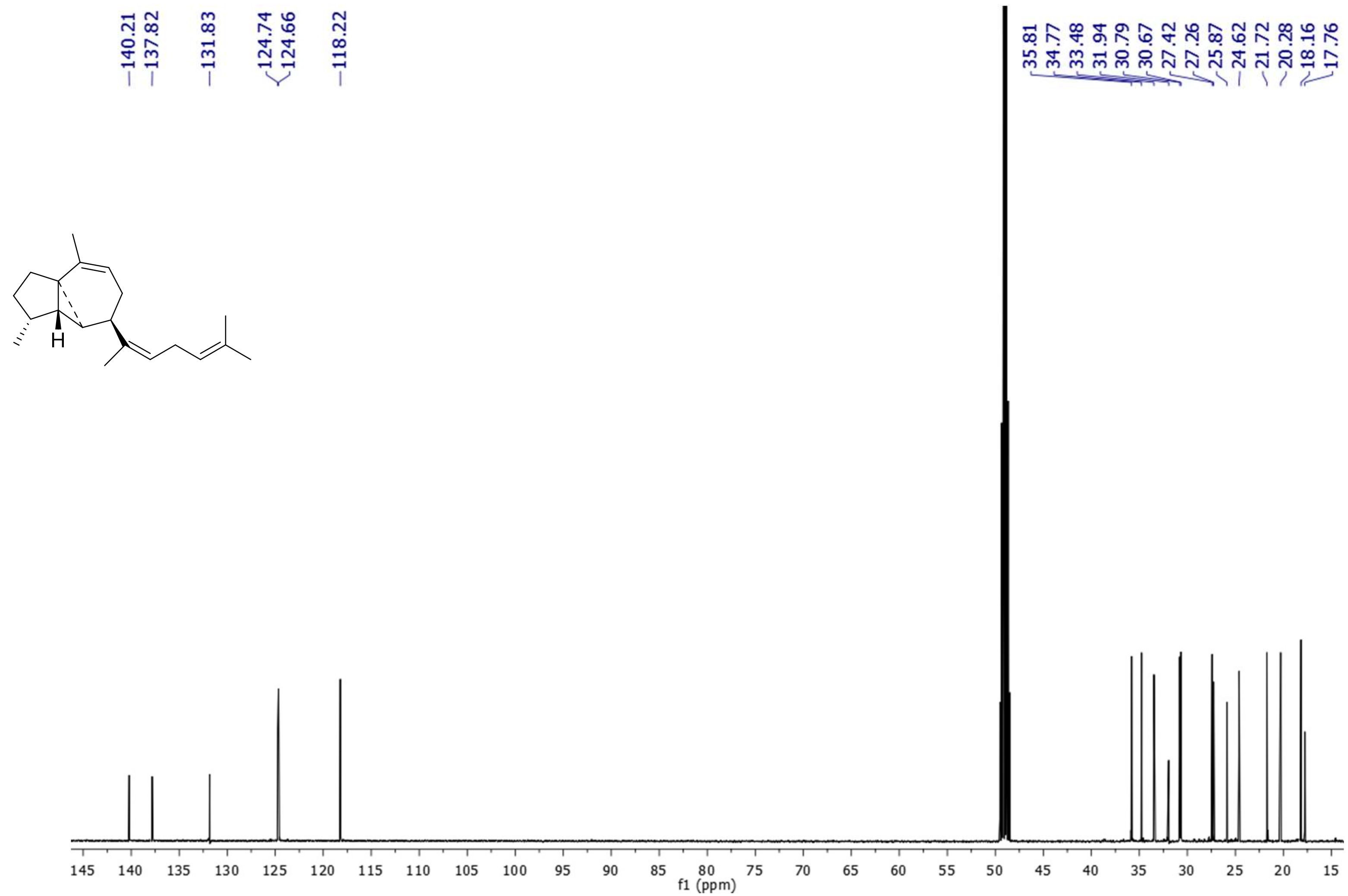

Figure S26. ^{13}C RMN spectrum (125 MHz, CD_3OD) of compound **15**

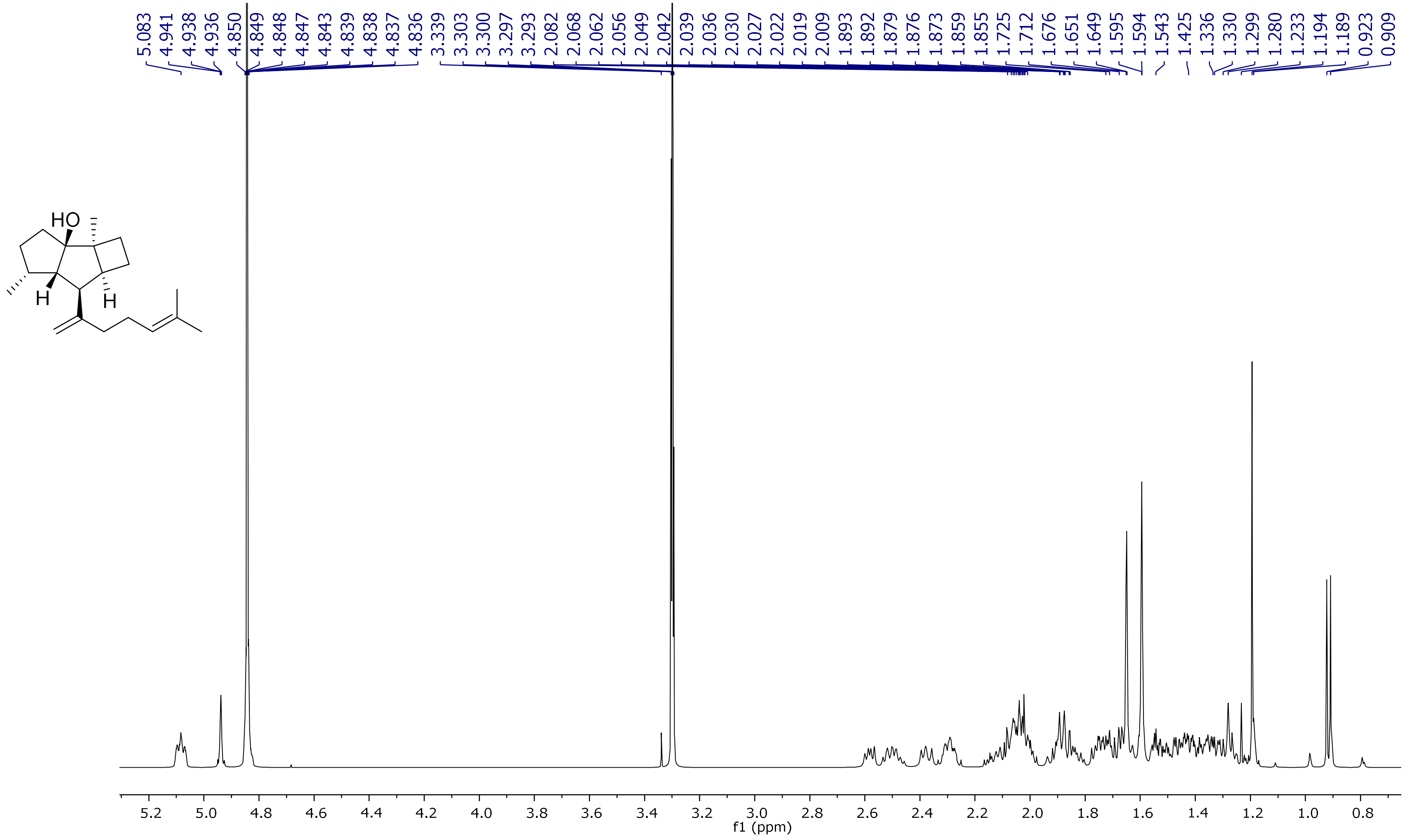

Figure S27. ^{1}H RMN spectrum (500 MHz, CD_3OD) of okamurol A (**16**)

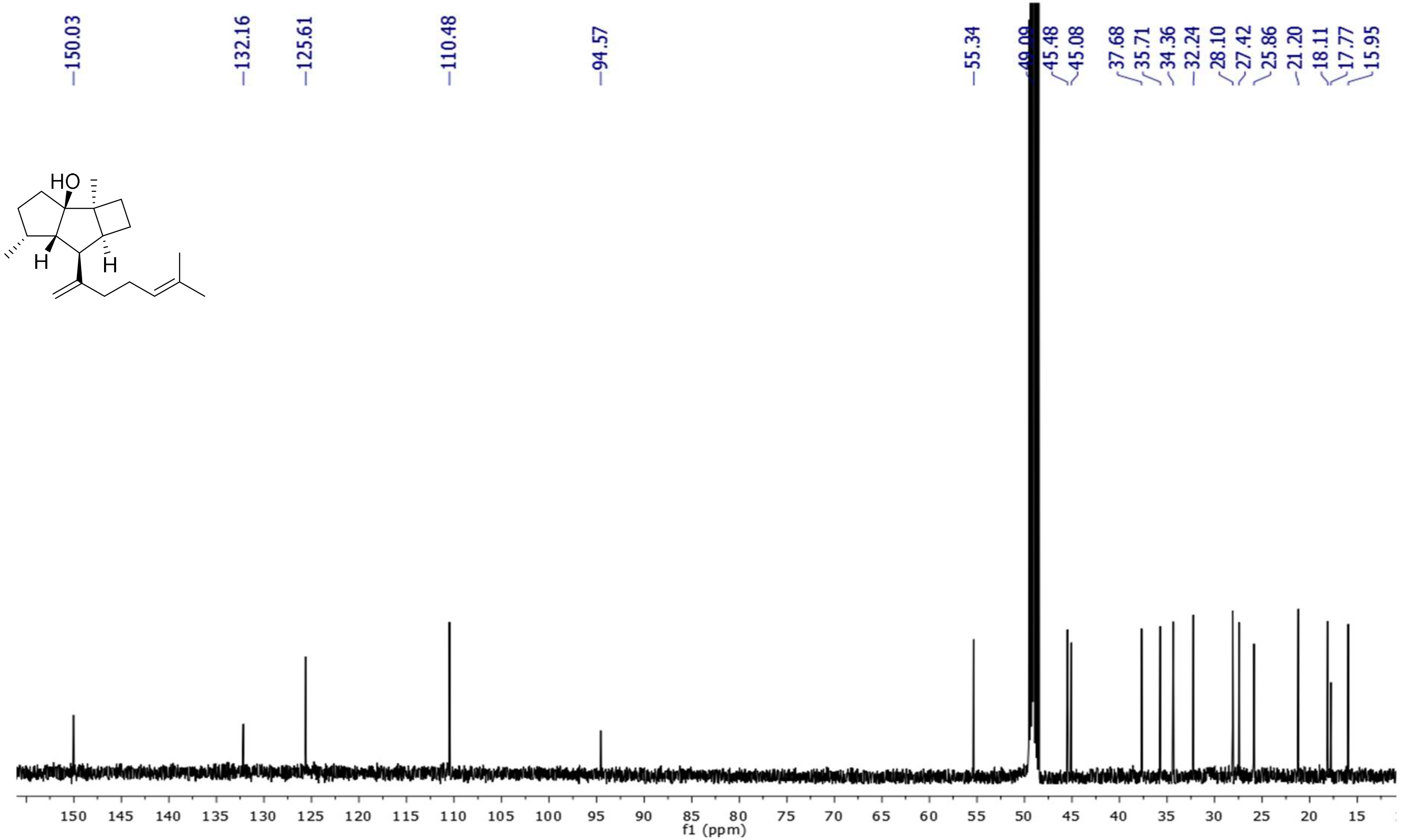

Figure S28. ^{13}C RMN spectrum (125 MHz, CD_3OD) of okamurol A (**16**)

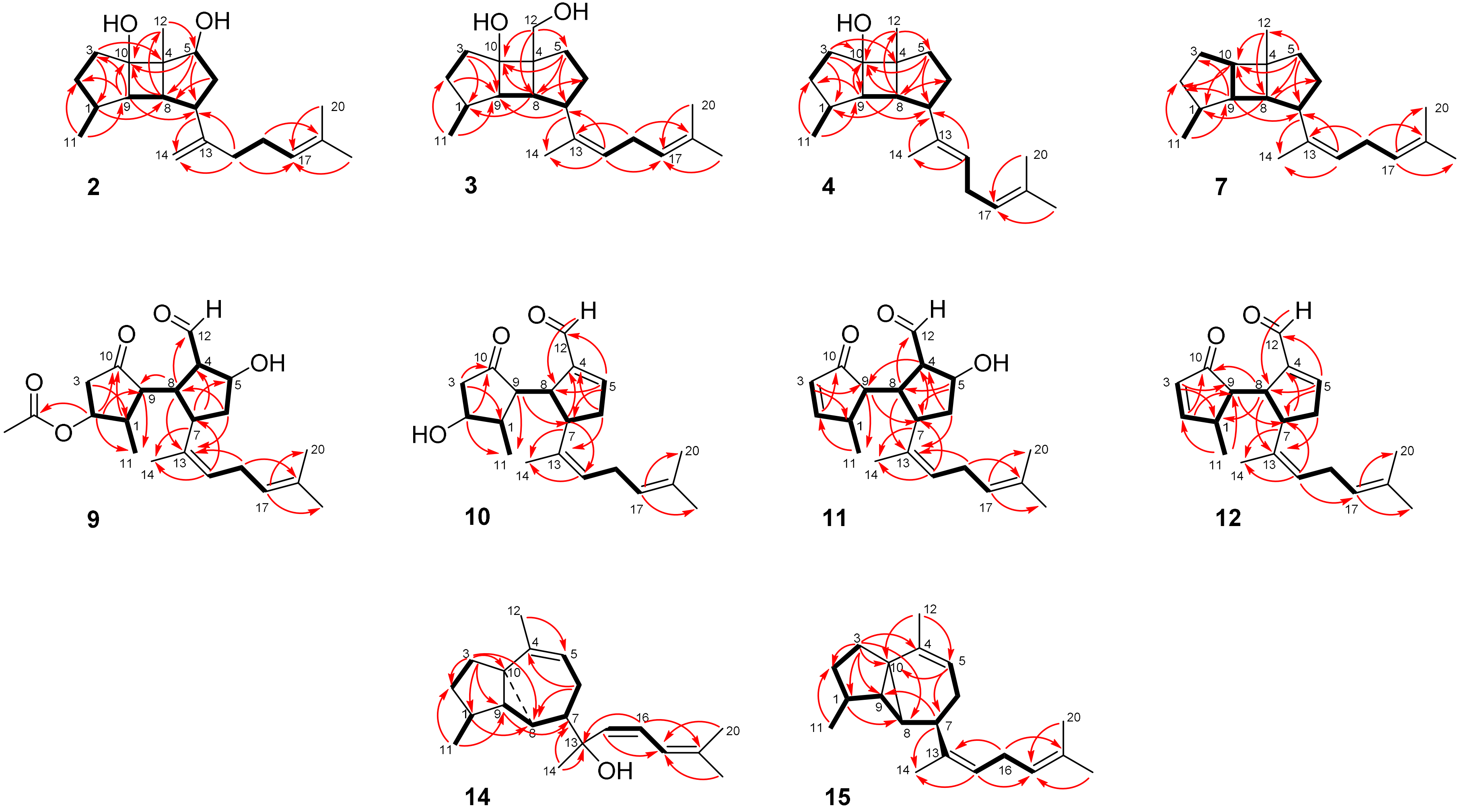

Figure S29. Key COSY (bold bond) and HMBC correlations (arrow) observed for compounds **2-4**, **7, 9-12**, **14**, and **15**

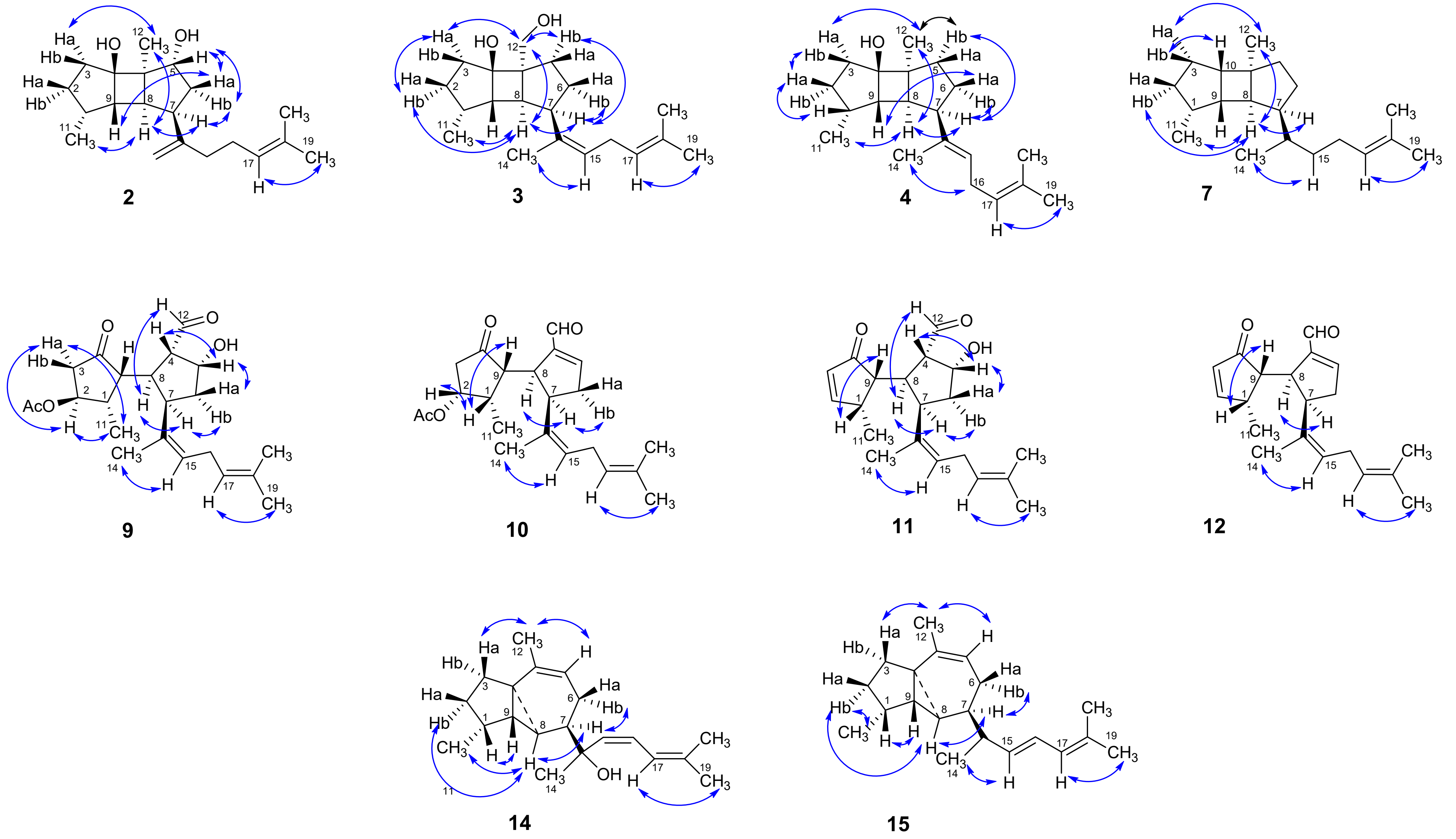

Figure S30. Key NOESY correlations observed for compounds **2-4**, **7**, **9-12**, **14**, and **15**

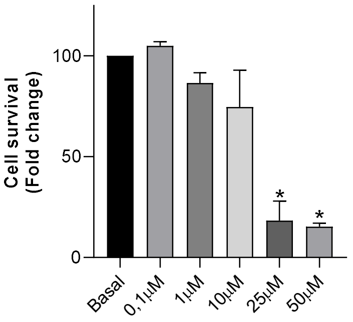

Compound **1**

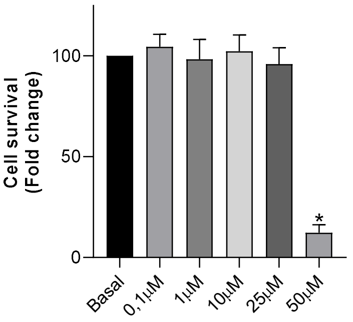

Compound **3**

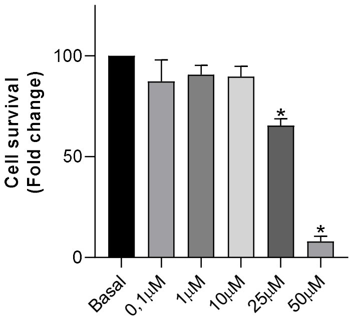

Compound **5**

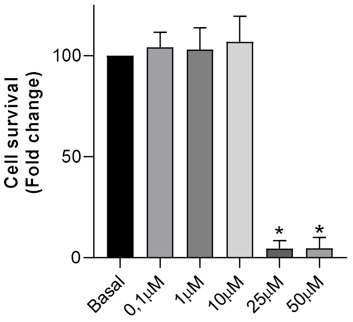

Compound **6**

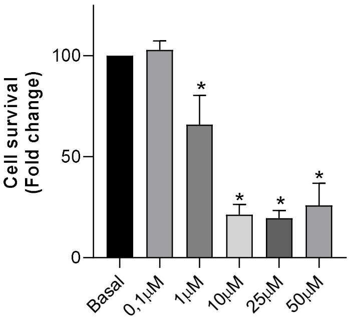

Compound **8**

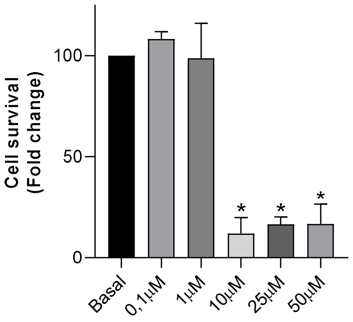

Compound **10**

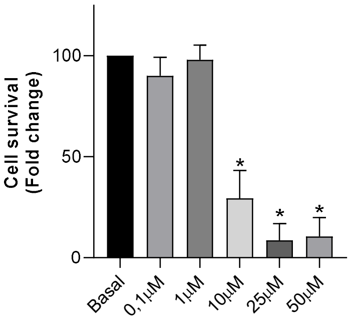

Compound **11**

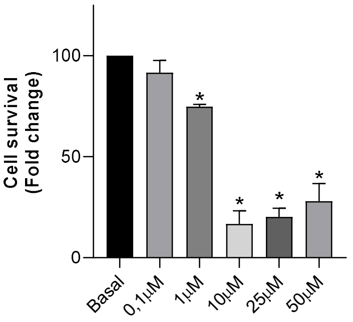

Compound **12**

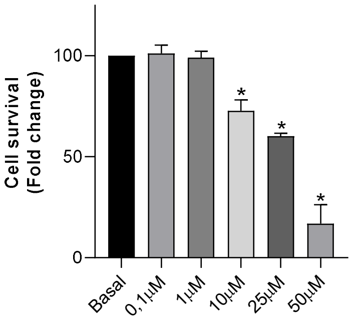

Compound **14**

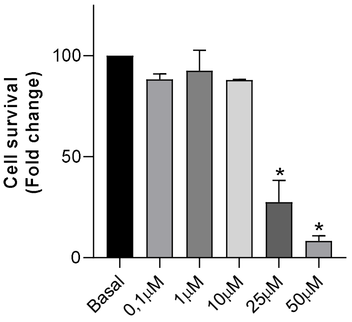

Compound **16**

Figure **S31.** Dose-response cell viability of Bv.2 cells was determined by crystal violet assay. The results are mean ± SD ($n \geq 3$ independent experiments performed in duplicate). Significant differences were determined by two way ANOVA followed by Bonferroni t-test *$p \leq 0.05$ vs Basal.

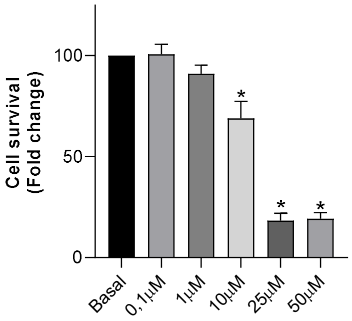

Compound **1**

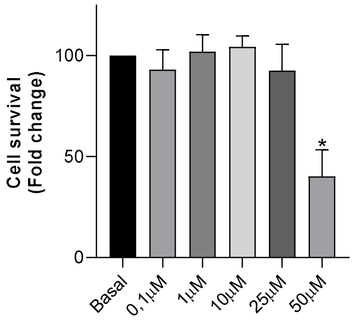

Compound **3**

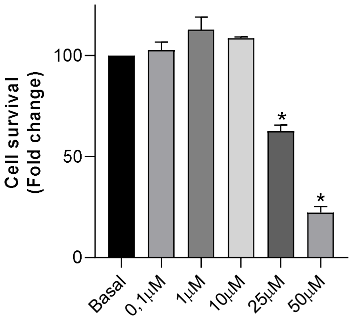

Compound **5**

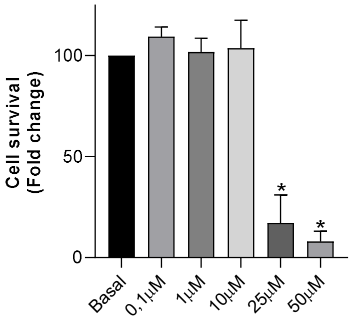

Compound **6**

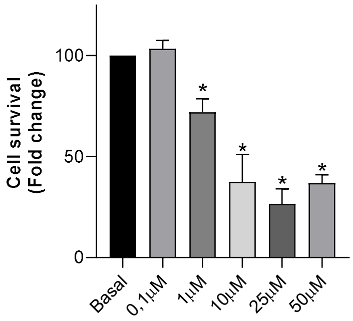

Compound **8**

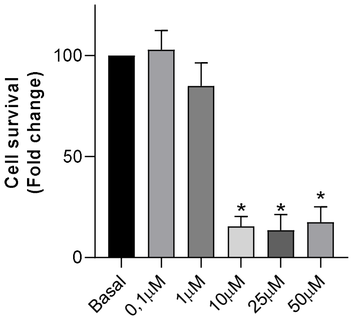

Compound **10**

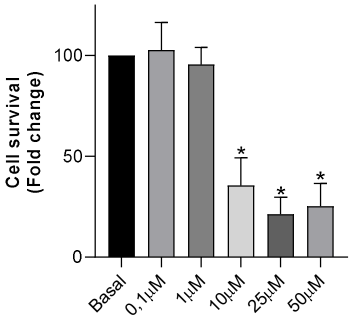

Compound **11**

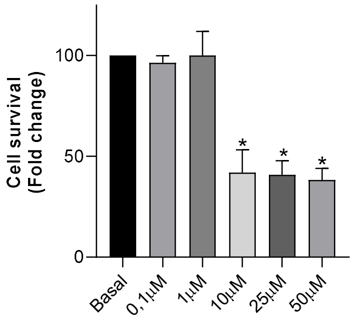

Compound **12**

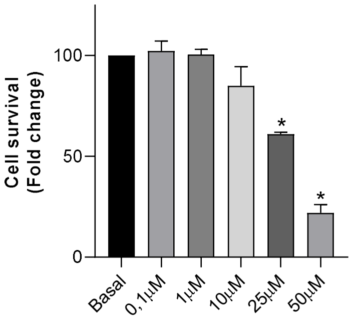

Compound **14**

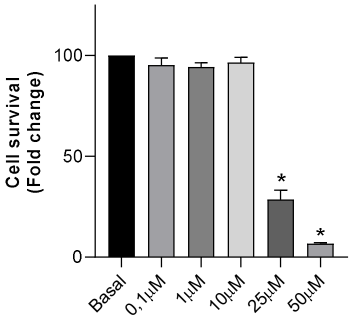

Compound **16**

Figure **S32.** Dose-response cell viability of RAW 264.7 cells was determined by crystal violet assay. The results are mean ± SD (n ≥ 3 independent experiments performed in duplicate). Significant differences were determined by two way ANOVA followed by Bonferroni t-test *$p \leq 0.05$ vs Basal.
